# Mechanisms underlying the low-temperature adaptation of 17β-estradiol-degrading bacterial strain *Rhodococcus* sp. RCBS9: insights from physiological and transcriptomic analyses

**DOI:** 10.3389/fmicb.2024.1465627

**Published:** 2024-11-21

**Authors:** Qiannan Li, Hanyu Pan, Peng Hao, Zhenhua Ma, Xiaojun Liang, Lianyu Yang, Yunhang Gao

**Affiliations:** ^1^College of Veterinary Medicine, Jilin Agricultural University, Changchun, China; ^2^Institute of Animal Science, Ningxia Academy of Agriculture and Forestry Sciences, Yinchuan, China

**Keywords:** *Rhodococcus* sp. RCBS9, degrading bacterium, low-temperature adaptation, transcriptome, 17β-estradiol

## Abstract

The 17β-estradiol (E2)-degrading bacterium *Rhodococcus* sp.RCBS9 previously showed remarkable resistance to the combined stresses of low temperature and E2. In this study, physiological experiments and transcriptomic analysis were performed to investigate the mechanisms underlying the strain's low-temperature adaptation and briefly analyze how it maintains its ability to degrade E2 at low temperature. The results showed that the strain's signal transduction functions, adaptive changes in cell membrane and cell wall structure, gene repair functions, and synthesis of antioxidants and compatible solutes are key to its ability to adapt to low temperature. In addition, its stress proteins in response to low temperature were not typical cold shock proteins, but rather universal stress proteins (USPs) and heat shock proteins (HSPs), among others. The strain also upregulated biofilm production, transporter proteins for carbon source uptake, and proteins for fatty acid degradation to ensure energy generation. The strain's multiple stress responses work synergistically to resist low-temperature stress, ensuring its adaptability to low-temperature environments and ability to degrade E2. Finally, six genes related to survival at low temperature (identified in the transcriptome analysis) were expressed in *E. coli* BL21, and they were found to contribute to recombinant *E. coli* growth at low temperature.

## Graphical Abstract

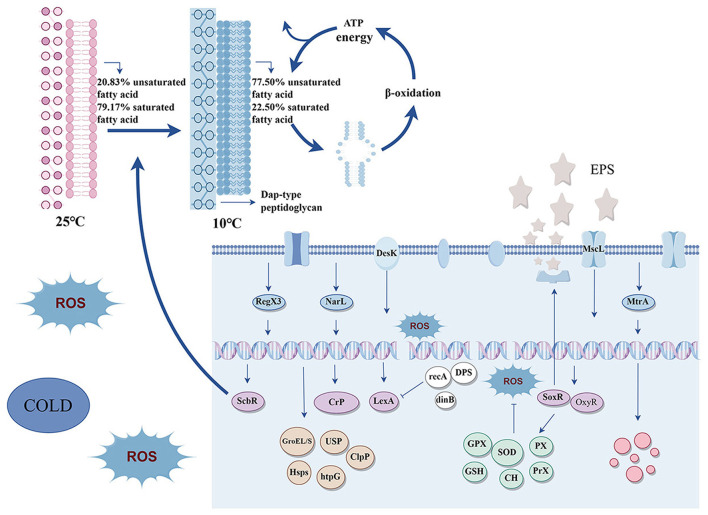

## 1 Introduction

Toxic pollutants, which are not easily broken down naturally in the environment, can pose a serious threat to various organisms in ecosystems (Bergmann et al., 2022; Kar et al., 2024; Yang et al., 2024). In addition to well-known pollutants such as plastics, pesticides, and antibiotics, emerging pollutants such as 17-β estradiol (E2) are of increasing concern (Adeel et al., 2017; Gao Y. et al., 2021). Estrogens have been found in water and soil in many parts of the world (Ciślak et al., 2023; Sacdal et al., 2020; Unnikrishan et al., 2024). They are persistent in the environment and can affect plant root and shoot development, as well as severely disrupting the immune and endocrine systems of animals and humans (Adeel et al., 2018; Li Y.-F. et al., 2023; Salla et al., 2024). Various treatments are currently used to degrade E2.

In particular, microbial E2 degradation has attracted much attention due to its low cost, ease of operation, and high degradation efficiency. However, the ambient temperatures in many parts of the world change with the seasons, and the low temperatures in winter are challenging for microbial degradation activities. The oxidative and osmotic stresses associated with low temperatures can damage microbial cell membranes, cell walls, and DNA, and directly inhibit microbial protein activity, affecting metabolic reaction rates and nutrient uptake (Lv and Cheng, 2022; Zou and Cheng, 2024). These factors seriously reduce the survival and degradation efficiency of the degrading microorganisms.

Studies have explored the low-temperature tolerance mechanisms of polycyclic aromatic hydrocarbon- and heterocyclic polycyclic aromatic hydrocarbon-degrading bacteria (Song et al., 2024), lignin-degrading bacteria (Wang et al., 2022a), and alkane-degrading bacteria (Gregson et al., 2020). According to these studies, the abilities of microorganisms to sense the external environment and build cell membranes, cell walls, and biofilms are key for them to resist low-temperature stress and ensure internal environment homeostasis (Bao et al., 2023; Yan and Xie, 2020). The synthesis of stress-regulating substances and gene repair proteins also helps to ensure internal environmental homeostasis and maintain metabolic processes (Jin et al., 2022; Pátek et al., 2021). Different bacterial strains have different adaptation strategies to low temperatures. Little research has been conducted on the mechanisms of low-temperature tolerance of strains that can effectively degrade E2 at low temperature, so it is vital to research these mechanisms.

Our group previously isolated bacterial strains and screened for strains with a strong ability to degrade E2 at low temperature (10°C) in low-nutrient conditions, and *Rhodococcus* sp. RCBS9 was identified (Hao et al., 2024), but its isolated proteins lost E2-degradation activity at low temperatures (10°C). We hypothesized that this was because the strain has excellent low-temperature adaptation mechanisms that enable it to maintain estrogen uptake, synthesize degradation enzymes, and maintain degradation enzyme activity at low temperatures.

The aim of this study was to explore how *Rhodococcus* sp. RCBS9 adapts to low temperature. We first evaluated the stress response of the strain to low temperature by determining the levels of reactive oxygen species (ROS) and 8-hydroxy-2′-deoxyguanosine (8-OHdG), as well as cell membrane permeability. Subsequently, we investigated the mechanisms underlying low-temperature tolerance from both physiological and molecular perspectives. Regarding the physiological perspective, we assessed the superoxide dismutase (SOD) activity, carotenoid content, biofilm content, and fatty acid species and proportions in strain RCBS9. At the molecular level, we used transcriptome sequencing to study transcription and validated it using RT-qPCR. We also created recombinant *E. coli* that expressed target proteins related to survival at low temperature (identified in the transcriptome analysis), which were then grown at 10°C to explore whether the target proteins helped the *E. coli* to grow at low temperature. The results of this study will help us better use *Rhodococcus* sp. RCBS9 for E2 degradation in regions with varying temperatures. Additionally, our research contributes to the growing body of knowledge about bacteria that can degrade molecules at low temperatures, which will be useful for studying similar microorganisms.

## 2 Materials and methods

### 2.1 Bacterial culture

We used 10°C as the experimental condition to match our previous study (Hao et al., 2024). As 25°C is the optimal growth temperature of strain RCBS9, it was used as the control condition to fully understand the low-temperature tolerance mechanisms.

Strain RCBS9 (which was preserved in our laboratory) was streaked on LB agar plates and placed in a 25°C incubator to activate it. The activated strain was incubated at 10 or 25°C in liquid LB medium or MSM medium (containing minimal nutrients) supplemented with 10 mg/L E2. The experiments in Sections 2.2–2.6 and 2.9 were conducted in both LB and MSM+E2 media. The experiments in Sections 2.7, 2.8, 2.10, and 2.11 were conducted only in LB medium. The medium composition was as described by Hao et al. (2024).

### 2.2 Cell membrane permeability determination

The change in cell membrane permeability of strain RCSB9 in response to a reduction in temperature was determined by two methods: crystal violet staining (Devi et al., 2013; Herndon et al., 2020) and conductivity measurements (Kong et al., 2024). The strain RCBS9 was cultured to the logarithmic growth phase at 25°C and then incubated at 10°C for 2, 24, 48, and 72 h. Samples were collected at each time point to be used in both experiments. For the crystal violet assays, strain RCBS9 samples were mixed with crystal violet (Aladdin Biochemical Technology Co. Ltd., Shanghai, China), incubated at 37°C for 10 min, and centrifuged (Eppendorf, Germany) at 13,500 rpm, and the optical density (OD) at 590 nm of the supernatant was measured using a microplate reader (Thermo Fisher, Swedish). For the conductivity assays, strain RCBS9 samples were centrifuged at 8,000 rpm for 5 min, and the conductivity of the supernatants was then measured using a conductivity meter (Shanghai Yidian Scientific Instrument Co., Ltd., China).

### 2.3 ROS and DNA damage determination

The ROS levels and DNA damage of strain RCBS9 at 10 and 25°C were quantified using a ROS detection kit (Beyotime Biotechnology) and an 8-OHdG ELISA kit (Sangon Biotech), respectively. The results were determined using a fluorescence microplate (Shimadzu, Japan) and measured using a microplate reader (Thermo Fisher, Swedish), respectively.

### 2.4 SOD activity determination

SOD activity in strain RCBS9 at 10 or 25°C was determined using a SOD assay kit (Nanjing Jiancheng Bioengineering Institute). Total protein in strain RCBS9 was quantified using a bicinchoninic acid (BCA) protein content assay kit (Beyotime Biotechnology). SOD activity was then calculated using the formula provided in the SOD assay kit. The results were determined using a measured using a microplate reader (Thermo Fisher, Swedish).

### 2.5 Carotenoid content determination

Carotenoid content was assessed as described previously (Flores et al., 2020; Liu et al., 2020). RCBS9 suspension was transferred to a brown centrifuge tube (protected from light), mixed with lysozyme, incubated at 37°C for 30 min to lyse the bacteria, mixed with acetone for 5 min, and centrifuged (Eppendorf, Germany). The values at OD480 nm of the supernatant was assessed using a microplate reader (Thermo Fisher, Swedish). The carotenoid content (C) was calculated according to the following formula (the extinction coefficient $\varepsilon_{1cm}^{1\%}$ was set at 1,600 g%^−1^ cm^−1^): $C\;\left(ug/ml\right)=\left(A^{480}\times 10^{6}\right)/\left(e_{1cm}^{1\%}\;\times 100\right)$.

### 2.6 Biofilm content determination

Biofilm content was assessed as described previously (Yan and Xie, 2020). The cultured strain RCBS9 was placed in 12-well plates and incubated at 10 or 25°C for 48, 72, 96, or 120 h. The planktonic cells in the wells were aspirated and measured at OD600 nm using a microplate reader (Thermo Fisher, Swedish). Planktonic cells remaining in the wells were rinsed away with phosphate-buffered saline and the wells were air-dried to fix the adherent cells in the wells. Subsequently, 2 mL of 0.1 mg/mL crystal violet was added for 20 min to ensure complete attachment to the biofilm on the well surface. After rinsing away the excess dye and air-drying for fixation, 2 mL of 95% ethanol was added to solubilize the biofilm, and then the biofilm was quantified at OD590nm using a microplate reader (Thermo Fisher, Swedish). The relative biofilm content was determined based on the ratio of biofilm content to bacterial density.

### 2.7 Fatty acid content determination

Strain RCBS9 was cultured in LB medium at 10 or 25°C and processed according to the standard protocols of the MIDI/Hewlett-Packard Microbial Identification System (Keystone Laboratories, Edmonton, Canada; Song et al., 2008; Wang et al., 2022a). The fatty acid species and their proportions in the cell membrane were analyzed by gas chromatography-mass spectrometry (GC-MS) using an Agilent 7890B-5977B system (Agilent Technologies, Inc.).

### 2.8 RNA sequencing and analysis

Strain RCBS9 was cultured in LB medium at 10 or 25°C and stored at −80°C. The bacterial samples were then sequenced by Beijing Novozymes Technology Co. A probe was used to remove ribosomal RNA (rRNA) from the total RNA to enrich the mRNA. Standardization and statistical modeling were applied to calculate the *p*-values and then adjust for multiple testing (controlling the false discovery rate). Genes with fold change ≥2 and *p*_adj_ < 0.05 were classified as upregulated or downregulated differentially expressed genes (DEGs). ClusterProfiler software was used for Gene Ontology (GO) and Kyoto Encyclopedia of Genes and Genomes (KEGG) enrichment analyses of the DEGs. String protein interaction database and cytoscape software were used for Protein-protein interaction analysis of DEGs from transcriptome analysis results.

### 2.9 RT-qPCR

The accuracy of the transcriptome data was verified by RT-qPCR (Zhou et al., 2022). The primer sequences (Supplementary Table 1) for selected genes were designed using SnapGene software. Samples of strain RCBS9 were prepared in LB and MSM+E2 media at 10 or 25°C, as previously described (Hao et al., 2023). Sample RNA was extracted using a bacteria total RNA isolation kit (Sangon Biotech). 16S RNA expression served as a calibration reference. Amplification was detected using a LightCycler^®^ 96 system. The RT-qPCR data were analyzed using the 2^−ΔΔCT^ method to determine fold changes in gene expression.

### 2.10 Recombinant *E. coli* construction

Primers for six target genes in strain RCBS9 related to survival at low temperature (identified in the transcriptome analysis), i.e., sHsps, DPS, GroEL, USP-1, Cu/Zn-SOD, and USP-2, were designed by Sangon Biotech (Shanghai; Supplementary Table 2). The genes were amplified by PCR and the PCR products were purified using a DiaSpin DNA Gel Extraction Kit (B110092-0100; Sangon Biotech) before being cloned into a pET-28a(+) plasmid. The constructed plasmids and empty plasmid were then transformed into *E. coli* BL21 (DE3) cells (Deng et al., 2014; Li R. et al., 2023).

### 2.11 Sodium dodecyl sulfate-polyacrylamide gel electrophoresis and recombinant *E. coli* growth at low temperature

Proteins in the abovementioned recombinant *E. coli* were analyzed by SDS-PAGE. Recombinant *E. coli* (BL21 pET-28a–target gene) and the control (BL21 pET-28a) were induced with identical concentrations of isopropyl β-D-thiogalactopyranoside (IPTG) at 16°C for 17 h to overexpress the fusion proteins (His–target protein) or His, respectively. Protein expression was then assessed by SDS-PAGE.

To monitor growth, after induction, the recombinant E. coli were resuspended in fresh LB medium, and the OD600 nm of the bacterial solution was adjusted to 1.0. The culture was then incubated at 10°C for 0, 4, 6, 8, and 24 h, with measurements of bacterial growth (OD600 nm) recorded at each time point (Xikeranmu et al., 2020).

### 2.12 Statistical analysis

Data were collated using Excel 2019 and analyzed (plotting graphs and conducting *t*-tests and one-way ANOVA) using GraphPad Prism 8.0. All experiments were performed in triplicate, and the results were expressed as means with standard deviations (ns, not statistically different; ^*^P < 0.05, ^**^P < 0.01, ^***^P < 0.001, ^****^P < 0.0001).

## 3 Results and discussion

### 3.1 ROS levels and DNA damage

**Figure 1:**
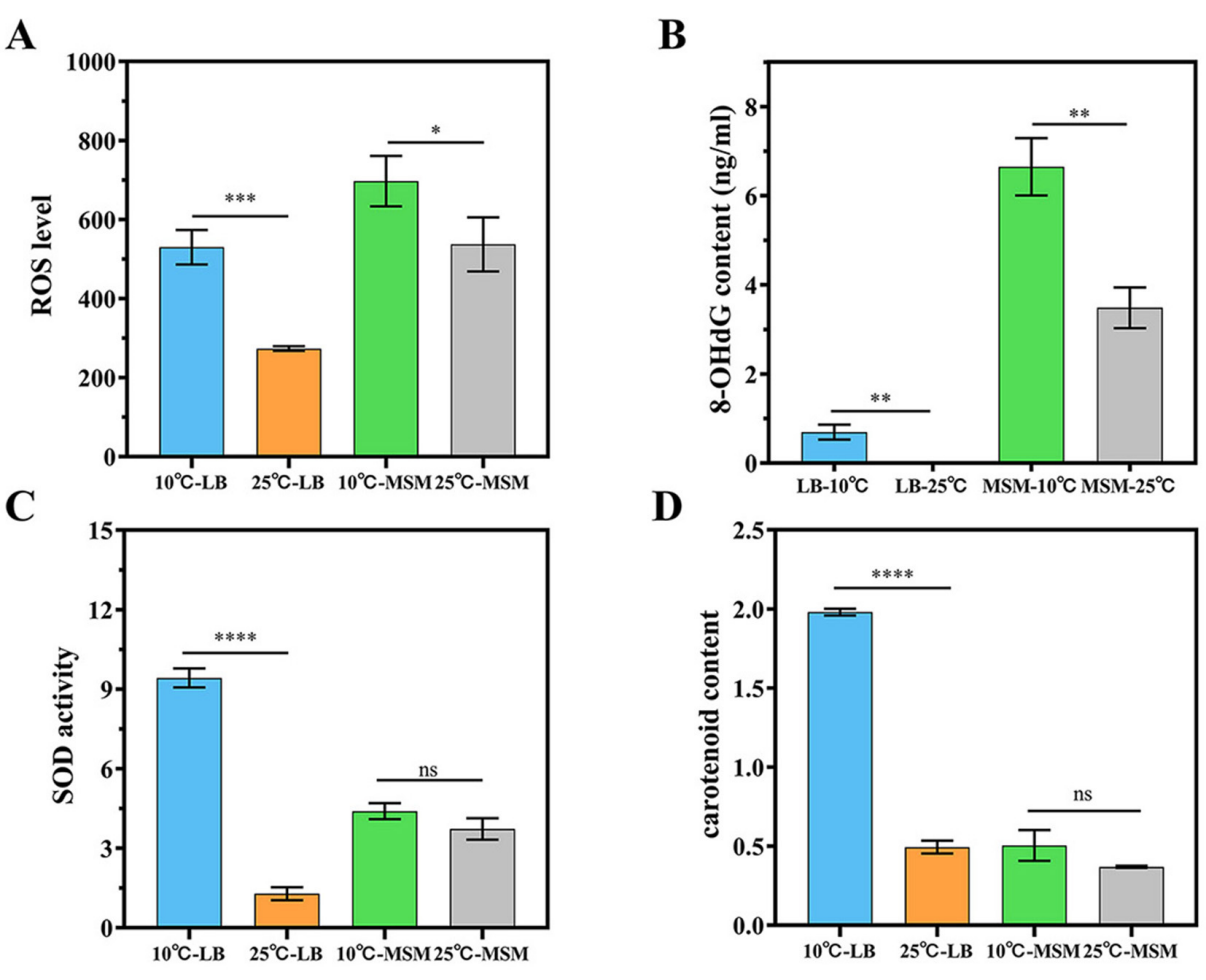
Physiological properties of strain RCBS9 at 10 and 25°C. **(A)** ROS levels in strain RCBS9 at 10 and 25°C. **(B)** 8-OHdG levels in strain RCBS9 at 10 and 25°C. **(C)** SOD activity. **(D)** Carotenoid content. ns, not statistically different; *P < 0.05, **P < 0.01, ***P < 0.001, ****P < 0.0001.

Although strain RCBS9 grew and degraded >70% of E2 at 10°C, its growth and degradation efficiency were significantly decreased at this low temperature compared to at 25°C (Hao et al., 2024). To elucidate the reasons for the decreases at low temperature, we measured the levels of ROS and 8-OHdG [a marker of ROS-induced DNA damage (Shan et al., 2020)] in the strain. The results showed that ROS and 8-OHdG were significantly higher at 10°C than 25°C (Figures 1A, B). At low temperatures, the increased oxygen solubility leads to ROS accumulation, causing oxidative damage to cellular biomolecules, with ROS levels serving as an indicator of bacterial stress (Irshad et al., 2021; Zou and Cheng, 2024). For instance, ROS levels have been used to assess the stress response to Fe_3_O_4_ nanoparticles in anaerobic ammonium oxidation microbial communities (Zhang et al., 2024). The experimental results showed that strain RCBS9 was subjected to oxidative stress at low temperature and the DNA was attacked by ROS, causing genetic damage and thus reducing the growth rate and E2 degradation capacity of strain RCBS9 at low temperature. Additionally, ROS and 8-OHdG levels were higher in MSM+E2 medium than in LB medium, likely due to the additional oxidative stress due to E2 (Chainy and Sahoo, 2020) and the nutrient-poor environment in MSM+E2 medium (Husain et al., 2022).

### 3.2 SOD activity and carotenoid content

**Figure 2:**
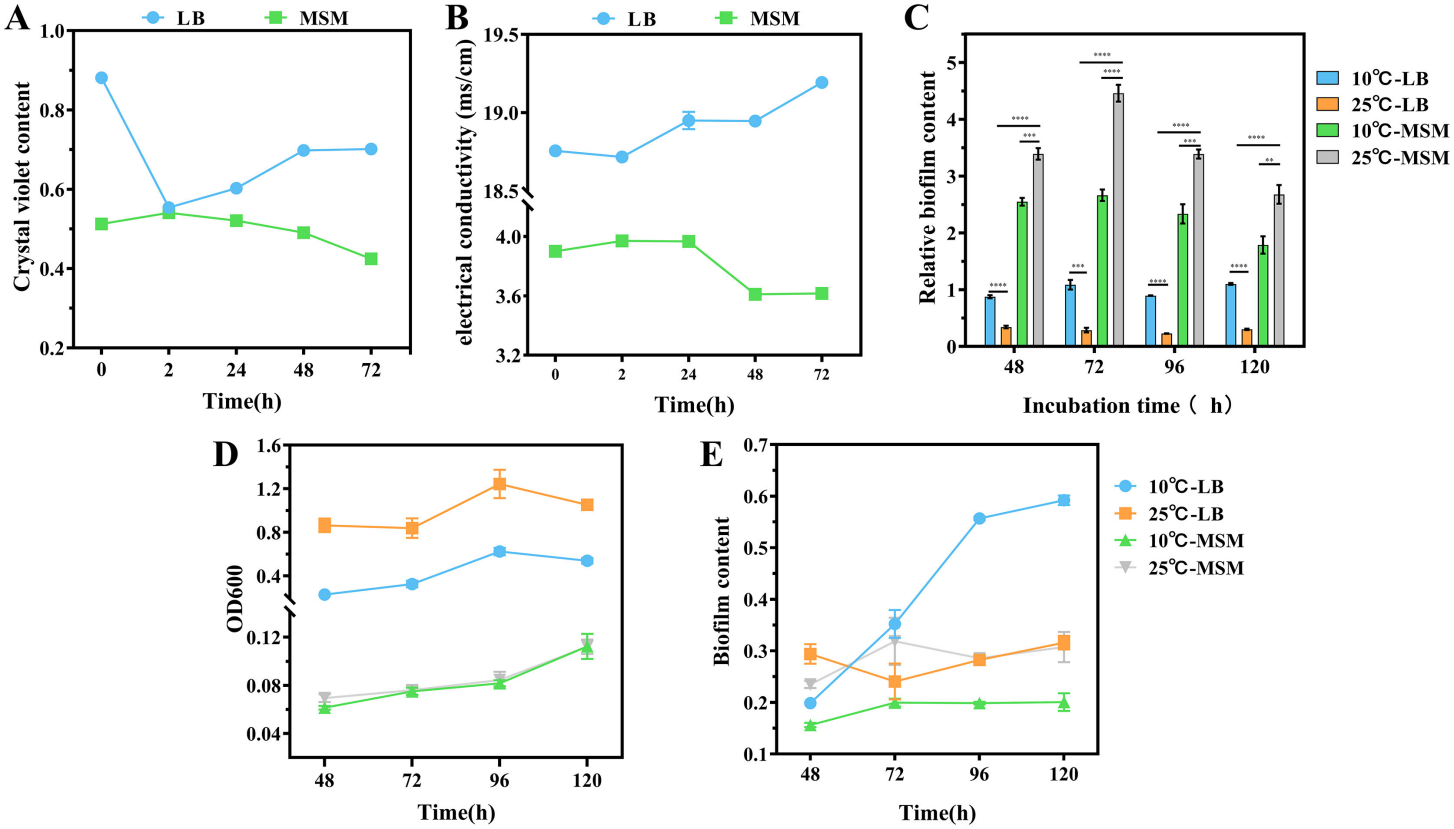
Cell membrane permeability of strain RCBS9 at 10 and 25°C, based on **(A)** crystal violet content and **(B)** electrical conductivity values. **(C)** Relative biofilm content (ratio of biofilm content to the value at OD600 nm for bacteria). **(D)** Bacterial load during biofilm production (value of bacterial solution at OD600 nm). **(E)** Biofilm content. ns, not statistically different; *P < 0.05, **P < 0.01, ***P < 0.001, ****P < 0.0001.

This study aimed to examine whether strain RCBS9 withstands low temperatures by producing SOD and carotenoids. The experimental results showed that Figures 2A, B, strain RCBS9 in LB medium had significantly higher SOD activity and carotenoid content at 10°C than at 25°C. Strain RCBS9 can scavenge ROS accumulated at low temperatures by increasing SOD activity and carotenoid content (Chen et al., 2023; Shi et al., 2022; Yu Z. et al., 2023), thus avoiding excessive damage or even death of the bacterium caused by high ROS levels and ensuring its growth and metabolism under low temperature conditions. Carotenoids also increase cell membrane stability (Chia et al., 2021), which is another assurance that strain RCBS9 can survive at low temperature. This phenomenon has also been observed in other cryotolerant bacteria (Flores et al., 2020; Li et al., 2022). In MSM+E2 medium, there was also higher SOD activity and carotenoid content at 10°C than at 25°C, but the differences were not significant (Figures 1C, D). The presence of E2 induces oxidative stress, which may alter intracellular SOD activity and carotenoid content, so additional low-temperature stress did not significantly modulate them. Taking the results of the two experiments together, under more severe conditions (low temperature and MSM+E2 medium), strain RCBS9 preferred to produce SOD rather than carotenoids to counteract low-temperature stress.

### 3.3 Cell membrane permeability

Low temperature affects the fluidity of the phospholipid bilayer, which is a part of the bacterial cell membrane, and membrane protein activity (Li et al., 2020). This affects cell membrane permeability and inhibits bacterial growth and reproduction. Therefore, the ability to maintain cell membrane function at low temperature is crucial for cold-tolerant bacteria. To examine the functional status of the cell membrane of strain RCBS9 under low-temperature stress, the cell membrane permeability was assessed.

First, crystal violet staining showed that the cell membrane permeability of strain RCBS9 in LB medium was significantly reduced after 2 h at 10°C compared to at 25°C, which was consistent with findings regarding other bacteria (Li et al., 2020; Wang et al., 2022a). The permeability gradually increased over time at 10°C, which may reflect a gradual recovery of the cell membrane function. In MSM+E2 medium, the cell membrane permeability was increased at after 2 h at 10°C compared to 25°C. We hypothesize that because E2 was the only carbon source, the cell membrane construction in strain RCBS9 was incomplete. This phenomenon has also been observed in other bacteria (Salton, 1967). The subsequent decrease in permeability over time may be due to strain RCBS9 restoring the cell membrane structure and function as it adapted to its environment.

Second, conductivity assays were carried out (in both LB and MSM+E2 media) during the bacterial growth plateau, to avoid errors due to increased bacterial counts. The results followed similar trends to those of the crystal violet assays. The only significant difference between the two experimental trends was that the conductivity of the bacterial fluid at 72 h in LB medium was significantly higher at 10°C than at 25°C, which may be due to the accumulation of metabolic by-products from prolonged incubation in a nutrient-rich environment.

Overall, the changes in cell membrane permeability of strain RCBS9 are a combination of the direct effects of low temperature on the cell membrane and the restoration of bacterial adaptive capacity over time. The recovery of cell membrane function over time is crucial for the survival of strain RCBS9, but the specific mechanisms have yet to be studied in depth.

### 3.4 Relative biofilm content

A bacterial biofilm is a structured community of bacteria that adheres to a solid surface by secreting extracellular polymeric substances. These substances provide a physical protective barrier for the bacteria, as well as trapping nutrients while eliminating waste (Camba et al., 2024; Kuttel et al., 2017; Yan and Xie, 2020). We investigated whether the relative biofilm content of strain RCBS9 (defined as the ratio of biofilm content to the value at OD600 nm for bacterial solution) increased at low temperature. The relative biofilm content in LB medium was consistently higher at 10°C than at 25°C, suggesting that strain RCBS9 resists low temperature by producing biofilm. Notably, the relative biofilm content was significantly higher in MSM+E2 medium than LB medium. This phenomenon should indicate that, in nutrient-limitation stress (at 25°C in MSM), strain RCBS9 formed biofilms to capture carbon-E2 (Du et al., 2022). Additional low-temperature stress (at 10°C in MSM) did not increase the relative biofilm content compared to that at 25°C in MSM. Instead, the relative biofilm content decreased, which may be attributable to overwhelming stress (low-temperature stress plus nutrient-limitation stress; Figures 2C–E). In conclusion, the results demonstrated that biofilm production by strain RCBS9 is an effective adaptation strategy to low temperature and for capturing E2.

The aforementioned assays indicate that strain RCBS9 can undergo various adaptive changes to tolerate low temperature. Additionally, there was a degree of overlap in the strain's responses to low temperature, E2, and nutritional deficiency. When analyzing the strain's low-temperature tolerance mechanisms, the combination of multiple stress factors may lead to non-significant or even opposite-direction assay results. This could interfere with our comprehensive analysis of the low-temperature adaptive mechanisms of strain RCBS9. In order to gain a comprehensive understanding of the low-temperature response strategy of strain RCBS9 and to screen for effective low-temperature-tolerant protein, subsequent experiments were performed in LB medium with low temperature as the only variable to conduct in-depth fatty acid content analysis and transcriptome analysis.

### 3.5 Fatty acid content

**Figure 3:**
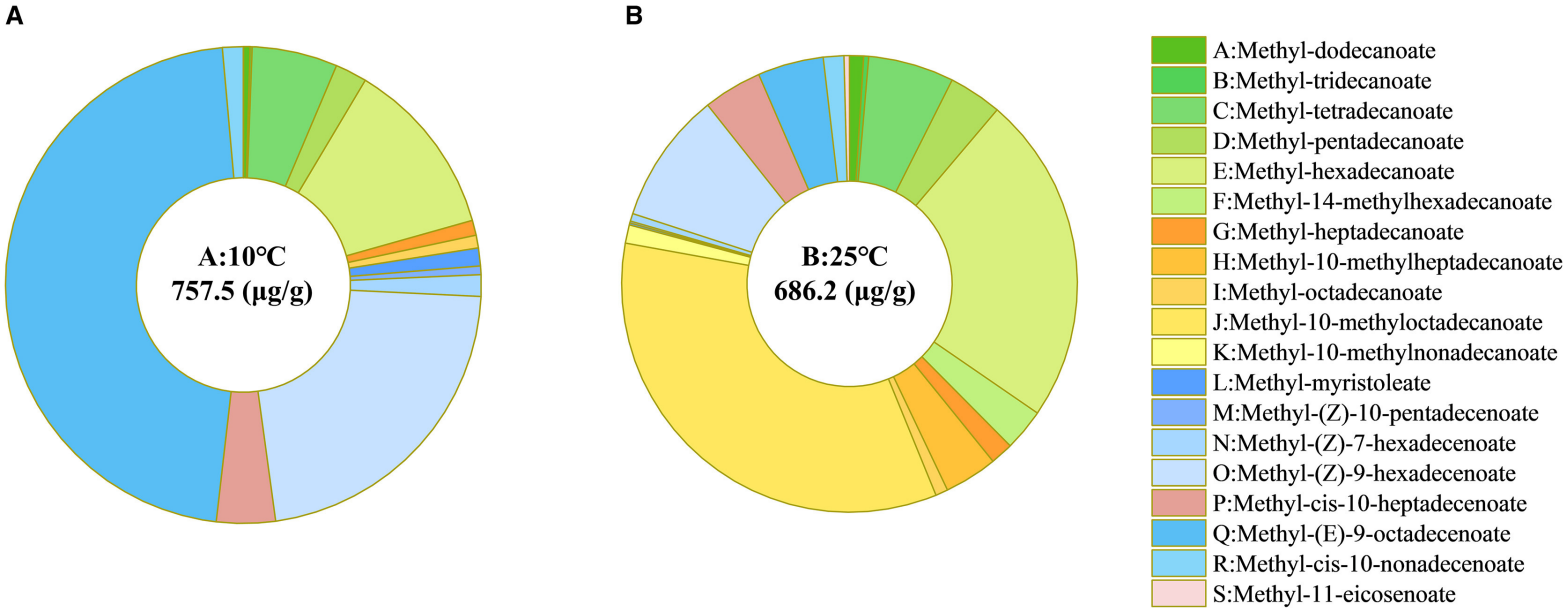
Fatty acid types and proportions in strain RCBS9 at **(A)** 10°C and **(B)** 25°C. The central value in each ring plot is the total amount of fatty acids at 10 or 25°C. Green represents C12–C16 saturated fatty acids. Yellow represents C17–C19 saturated fatty acids. Blue represents C14–C16 unsaturated fatty acids. Gray represents C17–C20 unsaturated fatty acids.

Fatty acids are integral components of cell membranes, and alterations in the types and proportions impact the fluidity and permeability of cell membranes (Yang et al., 2020). To determine the mechanisms underlying the cell membrane permeability changes in strain RCBS9 at low temperature and the adaptive measures employed by strain RCBS9 to regulate the composition of its cell membranes at low temperature, we investigated the fatty acid types and proportions in strain RCBS9 in LB medium at low temperature. The number of fatty acid species decreased from 19 at 25°C to 14 at 10°C. The content and proportions of saturated fatty acids also decreased at 10°C compared to 25°C. However, the overall fatty acid proportion increased by 10.39%, and the content and proportions of unsaturated fatty acids strongly increased, particularly regarding methyl (E)-9-octadecenoate, which increased by a factor of 11 (Figure 3).

At low temperature, the changes in fatty acids in strain RCBS9 indicated increases in the synthesis of long-chain unsaturated fatty acids. Similar changes have been observed in several bacterial strains (Almeyda et al., 2020; Wang Y. et al., 2020). The ability of unsaturated fatty acids (compared to saturated fatty acids) to maintain superior cell membrane fluidity at low temperatures (Ballweg et al., 2020) is likely to contribute to the gradual and limited increase in the cell membrane permeability of strain RCBS9. Furthermore, studies have demonstrated increased unsaturated fatty acid content in *Lactobacillus plantarum* LIP-1 under acid stress and *Listeria monocytogenes* under nutrient stress (E et al., 2021; Wang et al., 2023), which represents an effective bacterial adaptation to harsh environments. The capacity of strain RCBS9 to undergo adaptive alterations in cell membrane composition, thereby restoring cell membrane permeability at low temperature, may represent a pivotal factor influencing its capacity to uptake carbon sources at low temperature.

### 3.6 Transcriptomics analysis

**Figure 4:**
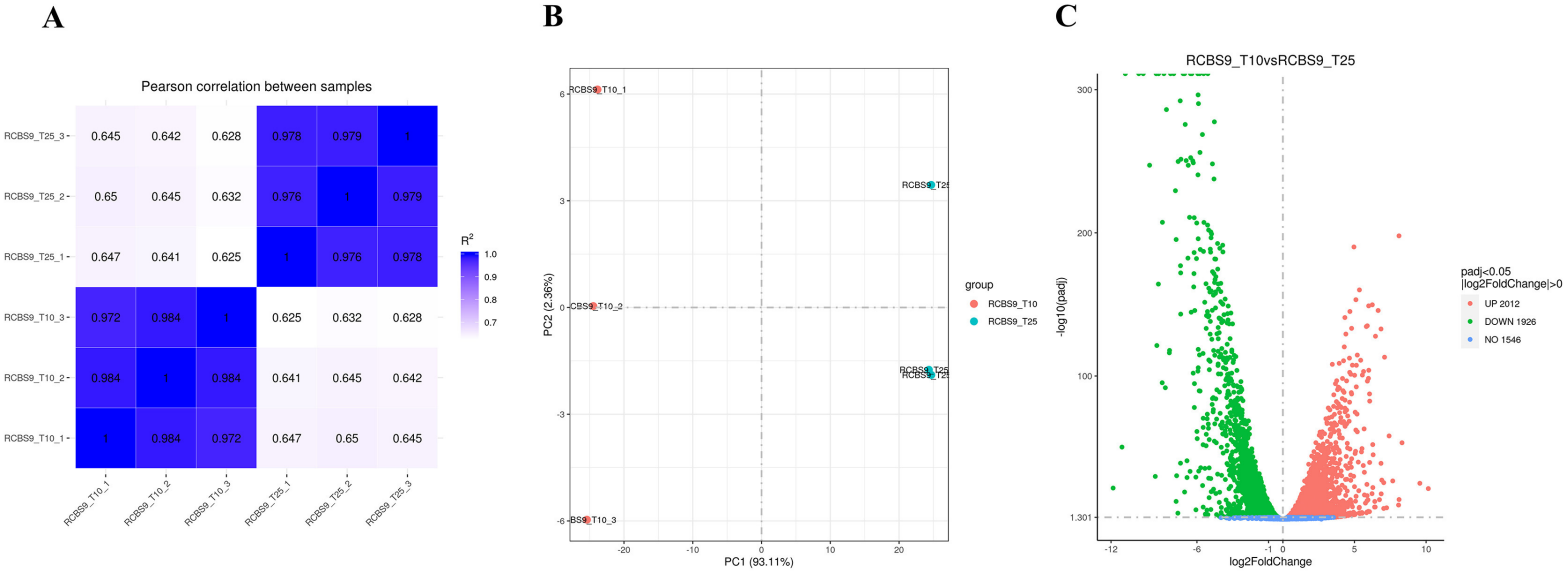
**(A)** Correlation plot of gene expression showing the squared correlation coefficients of each sample on the horizontal and vertical axes. **(B)** Principal component analysis plot of gene expression. The x- and y-axes display the first and second principal components, respectively. **(C)** Volcano plot. The x-axis displays log_2_(fold change), while the y-axis displays -log_10_p_adj_.

**Figure 5:**
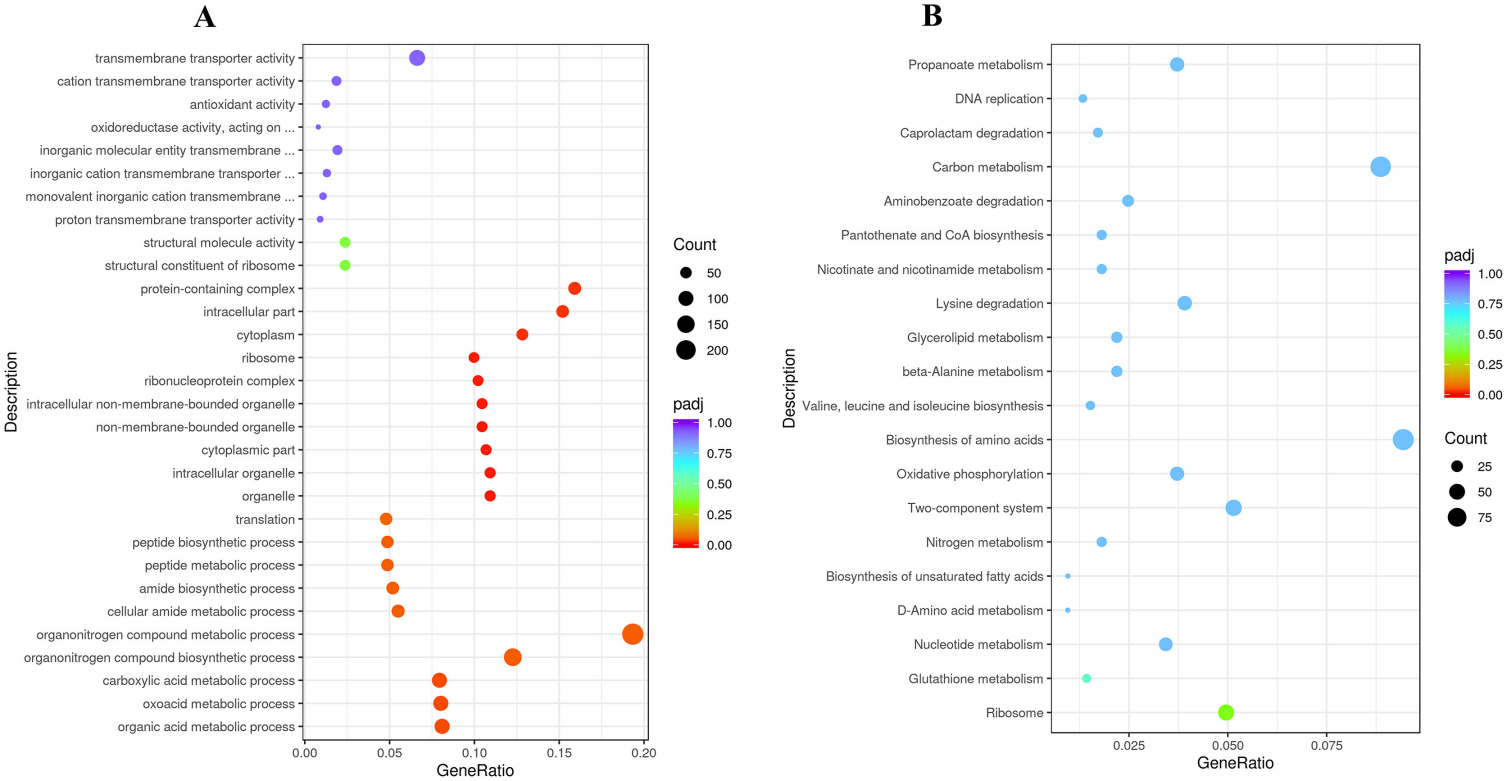
**(A)** GO and **(B)** KEGG enrichment analysis plots showing the top 20 enriched GO terms/KEGG pathways and the number of differentially expressed genes (DEGs) in strain RCBS9 at 10 vs. 25°C.

To gain a better understanding into how strain RCBS9 adapts to low temperature at a molecular level, we conducted transcriptome sequencing. Figure 4 shows a correlation plot of gene expression and Figure 5 shows a principal component analysis plot of gene expression, which confirm the reliability of the transcriptome data.

The volcano plot in Figure 4C reveals that there were 2,012 upregulated and 1,926 downregulated genes at 10 vs. 25°C. GO annotation of the DEGs (Figure 5A) showed that genes related to organic acid metabolism and ribosomes exhibited the most significant differences. Most of the upregulated genes were associated with localized transport and energy production, whereas most of the downregulated genes were linked to biomolecule synthesis. KEGG annotation of the DEGs (Figure 5B) indicated that genes related to secondary metabolite synthesis, metabolic pathways, transporters, two-component systems, and ribosomes exhibited the most significant differences. Most of the upregulated genes were associated with ABC transporter proteins, amino acid metabolism, and fatty acid degradation, whereas most of the downregulated genes were associated with ribosomes. These results suggest that strain RCBS9 requires more energy and transporter protein production to survive at 10°C compared to 25°C. The strain also reduces unnecessary protein synthesis.

The following sections discuss the transcription of cold adaptation-related genes at 10 vs. 25°C to provide insights into the regulation of the various stress response systems (Bao et al., 2023; Kloska et al., 2020; Pátek et al., 2021; Yan and Xie, 2020) of strain RCBS9 at low temperature.

#### 3.6.1 Signal transduction and transcriptional regulation

Bacteria living in complex environments tend to have an increased ability to detect and adapt to stimuli (Wiesmann et al., 2023; Zhao et al., 2023). One of the main reasons for the survival of strain RCBS9 in cold environments containing E2 should be its possession of complex signaling pathways that enable it to sense environmental changes and adjust its physiological processes accordingly. Cold stress activates strain RCBS9's histidine kinase DesK, a membrane-bound heat sensor, which transmits external signals to the cell to alter transcription, allowing cold adaption (Cybulski et al., 2010; Inda et al., 2019). We found that genes responsible for phosphate and nitrate/nitrite assimilation (RegX3 and NarL; Goddard et al., 2017; Park et al., 2019) and cell membrane stress and osmoprotection (MtrA; Hoskisson and Hutchings, 2006) were upregulated in strain RCBS9 at 10°C and that these genes are important for the survival of the strain at low temperatures. Secondary messengers also play a significant role in signaling processes. Recent research has demonstrated that cyclic dimeric guanosine monophosphate (c-di-GMP) increases biofilm formation at low temperatures (Lin et al., 2024). We found that some genes encoding enzymes involved in c-di-GMP synthesis were significantly up-regulated at 10°C. These transcriptional changes may be critical for bacterial survival at low temperature, allowing the bacteria to adapt more efficiently to their environment.

The genes of numerous global transcriptional regulators were upregulated at 10°C in response to external signals received by strain RCBS9. Among these, the Crp transcriptional regulators demonstrate an exceptional capacity to respond to a diverse array of intra- and extracellular signals (Körner et al., 2003; Krol et al., 2023). The LexA transcriptional regulators primarily regulate the SOS response of bacterial DNA (Fornelos et al., 2016; Kizawa et al., 2017). The OxyR and SoxR transcriptional regulators play pivotal roles in bacterial responses to oxidative stress and biofilm formation (Anand et al., 2019; Méndez et al., 2022; Wang et al., 2022b). The ScbR transcriptional activators are involved in bacterial quorum sensing and cell membrane formation (Wang et al., 2006). The upregulation of some of these transcriptional regulators in strain RCBS9 at 10°C regulated various processes (including oxidative stress responses, DNA and protein damage repair, cell membrane permeability, biofilm formation, and quorum sensing) through a complex regulatory network that responds to environmental changes and thereby optimizes survival and reproduction ability.

#### 3.6.2 Antioxidative stress systems

In response to oxidative stress at low temperatures, bacteria have evolved complex antioxidant defense systems to scavenge ROS and protect cells from oxidative damage (Borisov et al., 2021). All SOD genes were upregulated in strain RCBS9 at 10°C. This is consistent with the results of the SOD activity assays. Furthermore, peroxidase (PX), glutathione peroxidase (GPX), and peroxide reductase (Prx) genes were upregulated in strain RCBS9, while catalase (CAT) was downregulated. Low temperature causes ${\text{O}}_{2}^{-}$ accumulation in bacteria, and strain RCBS9 expresses SOD to convert the ${\text{O}}_{2}^{-}$ into O_2_ and hydrogen peroxide (H_2_O_2_; Zhao et al., 2021). H_2_O_2_ then needs to be promptly scavenged, and strain RCBS9 does this by upregulating PX, GPX, and Prx, but does not employ CAT, which specifically scavenges H_2_O_2_. Similarly, *Rhodococcus erythropolis* does not employ CAT to cope with oxidative stress at low temperatures (Wang C. et al., 2020). This may be because PX, GPX, and Prx can eliminate other peroxides unlike CAT, which can only degrade H_2_O_2_. Transcription translation and enzyme synthesis in the cell consume a large amount of energy, but the various metabolic pathways for energy production in bacteria are inhibited at low temperature. Thus, it is a prudent use of the limited energy to produce proteins with substrate and functional diversity. Strain RCBS9 also produces several antioxidants to scavenge ROS (Ghaedamini et al., 2023). Many genes encoding glutathione synthase and transporter enzymes were significantly upregulated in strain RCBS9 at 10°C, while genes encoding glutathione degradation enzymes were downregulated. Additionally, the enzyme associated with carotenoid synthesis, 4,4′-dimercaptoenal oxidase, was significantly upregulated, which is consistent with the results of the carotenoid content assays. The transcriptome data and the results of the SOD activity and carotenoid content assays clearly demonstrated that strain RCBS9 has a well-established and effective ability to scavenge ROS, which can help it to cope with the oxidative stress caused by 10°C and E2, helping strain RCBS9 to adapt to complex harsh environments for growth and reproduction.

#### 3.6.3 Maintenance of gene expression and repair of genes

ROS generated at low temperatures damages the genetic material of microorganisms, causing base mutations (8-OHdG formation) and DNA strand breaks. This is demonstrated by the elevated 8-OHdG content in strain RCBS9 at low temperature. This damage can be fatal for microorganisms (Han et al., 2023; Yu M. et al., 2023). Following DNA damage, cells activate coordinated responses including detection of DNA damage, blocking cell cycle progression and DNA replication, and repairing damage (Dubrez et al., 2020).

Many genes encoding enzymes required for gene expression processes in strain RCBS9 were downregulated at 10°C, while only a few were upregulated. This maintenance of gene expression processes only via a small number of proteins (which are presumed to work properly at low temperature) may have occurred because strain RCBS9 suffered DNA damage at 10°C and needed to reduce replication to avoid the transfer of damaged genes to daughter cells or to conserve energy in order to maintain life under harsh conditions (Chen et al., 2016). In addition, strain RCBS9 upregulated DNA starvation/fixed-phase protection protein (DPS) at 10°C to protect its gene sequences (Shahu et al., 2024).

DNA damage repair mechanisms represent a key factor for bacterial adaptation to harsh environments and survival. Strain RCBS9 possesses numerous genes encoding simple repair mechanisms, but only the genes for the base excision repair (BER)/mismatch repair (MMR) pathways, i.e., adenine-DNA glycosylase gene (mutY) (Wang et al., 2017), uracil-DNA glycosylase gene (UDG) (Chembazhi et al., 2017), and DNA glycosylase gene (mug; Yang et al., 2019) were upregulated at 10°C. Glycosylases are more specific regarding damage recognition and repair than nucleotide excision repair processes. Genes involved in DNA recombination repair and the SOS response (such as RecA, RecC, RecF, RecG, RecX, LexA, and dinB; Galhardo et al., 2009; Gao et al., 2023; Yakimov et al., 2017), were also highly upregulated at 10°C. The repair of DNA damage at low temperature using the small-scale but high-precision BER pathway and MMR pathway in strain RCBS9 is consistent with previous findings on bacterial DNA subjected to oxidative stress (Dubrez et al., 2020). DNA damage in strain RCBS9 at 10°C also initiated recombination repair and the SOS response together to maximize the integrity and correctness of the genetic material.

#### 3.6.4 Synthesis and degradation of cell membrane-related components

Strain RCBS9 exhibited significant alterations in the transcription of genes associated with the metabolism of substances related to cell membrane composition at 10°C. Genes encoding FabG enzymes (which are involved in the fatty acid synthesis pathway) exhibited a notable upregulation at 10°C. Conversely, genes encoding fatty acid desaturases (which are pivotal enzymes for unsaturated fatty acid synthesis) were downregulated. In addition, enzymes involved in fatty acid degradation such as β-oxidation-related enzymes and monooxygenases [which are also involved in amino acid metabolism and estrogen degradation (Sun et al., 2022)] were upregulated. Analysis of the protein-protein interaction network of all up-regulated expressed genes (Supplementary Figure 1), showed that the top 20 genes with the highest node degree were overwhelmingly associated with β-oxidation of fatty acids (Supplementary Table 7). This is not consistent with the fatty acid content assays (which showed that the proportions of unsaturated fatty acids strongly increased, while the proportions of saturated fatty acids decreased) or with studies of other cold-tolerant bacteria in which low temperatures usually downregulated fatty acid degradation genes and upregulated unsaturated fatty acid synthesis genes (Bao et al., 2023; Gao X. et al., 2021).

The results of the fatty acid content assays and transcriptomic analysis suggest that the increased fatty acid degradation by strain RCBS9 at low temperatures may be a low-temperature acclimatization strategy, and that strain RCBS9 obtains energy by recycling fatty acids, which distinguishes it from other cold-tolerant bacteria. Furthermore, many intermediates are generated during fatty acid degradation, and they play a crucial role in maintaining the stability of the intra- and extracellular environments. For example, the polymer of 3-hydroxybutyric acid (an intermediate of fatty acid degradation), polyhydroxybutyric acid, is closely related to biofilm formation (Tribelli and López, 2011), and the genes for key enzymes in polyhydroxybutyric acid synthesis were upregulated.

In a study on *E. coli*, it was found that translational regulation in response to environmental stress may result in a mismatch between mRNA and protein abundance (Zhang et al., 2022). This could be the reason for the mismatch between the transcription profile of fatty acid desaturases and the unsaturated fatty acid content in strain RCBS9.

It has been proposed that cell membrane lipids play an important role in the regulatory control of membrane-anchored proteins (Ristovski et al., 2021). We found that ABC transporter-related genes were highly upregulated at 10°C. Accordingly, strain RCBS9 may have increased the number of transporter proteins in the cell membrane for better nutrient uptake at low temperature.

#### 3.6.5 Synthesis and degradation of cell wall-related components

The cell wall is essential for bacteria to withstand osmotic pressure at low temperatures, maintain their shape, and stabilize the cell membrane. In strain RCBS9, certain genes involved in peptidoglycan synthesis were up-regulated at 10°C. The upregulated genes include those for DAP-type peptidoglycan synthetases, serine-type D-Ala-D-Ala carboxypeptidases, and peptidoglycan glycosyltransferases. DAP-type peptidoglycans strengthen the cell wall and make it more elastic, which helps bacteria to survive at low temperatures (Garde et al., 2021). Furthermore, the distinctive structural properties of DAP-type peptidoglycan render it resistant to certain antibiotics (Curtis et al., 1976), thereby indicating the resilience of strain RCBS9 to challenging environments and its capacity to degrade estrogens in many complex settings. The genes associated with converting L-amino acids to D-amino acids and UDP-N-acetylcytidylyl-L-alanine-D-glutaminase (involved in peptidoglycan synthesis; McGroty et al., 2013; Perdih et al., 2009) were also mostly all upregulated in strain RCBS9 at 10°C. Previous studies have demonstrated that certain D-amino acids influence peptidoglycan composition, amount, and strength by doping the polymer and regulating the enzymes that synthesize and modify the polymer (Cava et al., 2011; Dik et al., 2021; Miyamoto and Homma, 2021). The synthesis of DAP-type peptidoglycan and D-amino acids in order to construct cryogenic cell walls represents a crucial adaptation mechanism employed by strain RCBS9 to cope with the cryogenic environment.

#### 3.6.6 Stress-regulated protein expression

Numerous genes encoding universal stress proteins (USPs) were upregulated in strain RCBS9 at 10°C. These proteins expressed by strain RCBS9 at low temperature should play a role in sensing environmental changes, maintaining enzyme activity, and enhancing cell membrane stability (Fang et al., 2019; Yan et al., 2024). Genes encoding heat shock proteins (Hsps), including DnaK, htpG sHsp, DNAJ, and GRPE, were also upregulated in strain RCBS9 at 10°C. These proteins cooperate to form a dynamic and complex multifunctional network, which can regulate redox homeostasis (Smith et al., 2017; Velasco et al., 2019), maintain cytoplasmic protein homeostasis (Rosenzweig et al., 2019), and assist DNA damage repair (Dubrez et al., 2020). Notably, while genes related to Hsps were upregulated in strain RCBS9 at 10°C, a phenomenon that has been observed in other studies (Zhang et al., 2021), genes for cold shock proteins, which are commonly found during cold stress, were not upregulated. This differs from the low-temperature adaptation mechanisms of other bacteria, such as *Lactobacillus plantarum* LIP-1 (E et al., 2021). In addition, at 10°C, strain RCBS9 upregulated genes for chaperonins (GroES and GroEL), which help proteins to fold correctly and stabilize membrane lipids (Goltermann et al., 2016; Marchenkov et al., 2020), and genes for ClpP proteins, which degrade damaged proteins and control the central transcriptional regulators (Stahl et al., 2018; Xu et al., 2021). The ability of strain RCBS9 to upregulate a variety of stress-regulated proteins and proteins that maintain enzyme activity at low temperature contribute to its ability to survive at low temperature and to express E2-degrading enzymes and maintain their enzyme activity.

#### 3.6.7 Anti-osmotic stress systems

During low-temperature-related osmotic stress, in addition to two-component system sensors, mechanosensitive channels in bacterial cell membranes can sense cell membrane stretching due to water molecule movement as a result of high osmotic pressure (Zhao et al., 2020). Strain RCBS9 significantly upregulated its mechanosensitive channel protein, mscL, at 10°C. This enabled it to sense the osmotic condition of its growth medium and to regulate the flow of ion and water molecules, thereby alleviating osmotic pressure differences in the cell. The water channel protein genes of strain RCBS9 were also upregulated at 10°C. Many studies (Bremer and Krämer, 2019) have demonstrated that under high osmotic stress, the availability of free water is a fundamental determinant of bacterial growth, and the secondary effect of compatible solute accumulation, which increases volume, is key to its osmoprotective function. Compatible solutes prevent cell damage due to osmotic water loss and maintain cell expansion, which is essential for sustaining cell stretching, growth, and division (Bao et al., 2023; Sleator and Hill, 2002). Genes related to the transport of organic osmoregulators such as proline, glycine betaine, carnitine, choline, alginate, and maltose (e.g., ProP, OpuB, OpuC, OsmX, MalE, MalF, and SugB; Babu Sait et al., 2022; Culham et al., 2018; Druger-Liotta et al., 1987; Licht et al., 2019; Rath et al., 2020) were significantly upregulated in strain RCBS9 at 10°C. The gene responsible for betaine synthesis, betB (Zhu et al., 2022), was also upregulated. The genes for regulatory and transport proteins of K^+^ (KDP-ABCDE) were significantly downregulated. In most bacteria, K^+^ regulation is crucial for maintaining osmotic pressure (Stautz et al., 2021). However, the transcriptome data showed that the osmoprotective strategy of strain RCBS9 favored the accumulation of compatible solutes (e.g., glycine betaine and carnitine) over the accumulation of K^+^, which has been observed in studies of other Gram-positive bacteria (Rath et al., 2020; Song et al., 2020).

### 3.7 RT-qPCR verification of transcriptome results

**Figure 6:**
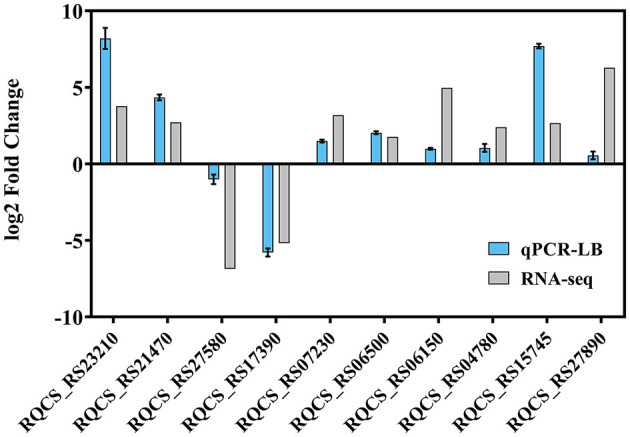
RNA-seq profiles of strain RCBS9 surviving at 10°C in LB medium were verified by RT-qPCR.

To verify the transcriptome results, RT-qPCR was performed to assess the transcription of strain RCBS9 in LB and MSM+E2 media. The RT-qPCR results of strain RCBS9 in the LB medium were completely consistent with the transcriptome results (Figure 6), which validated the transcriptome results. However, RT-qPCR results for two genes in MSM+E2 medium were inconsistent with the transcriptome results (Supplementary Figure 2). Combined with the results of the physiological assays described above, it can be assumed that the mechanisms of low-temperature tolerance of strain RCBS9 are similar under different conditions (LB medium vs. MSM+E2 medium), but they are appropriately adjusted to the specific environment.

### 3.8 Growth of recombinant *E. coli* at low temperature

**Figure 7:**
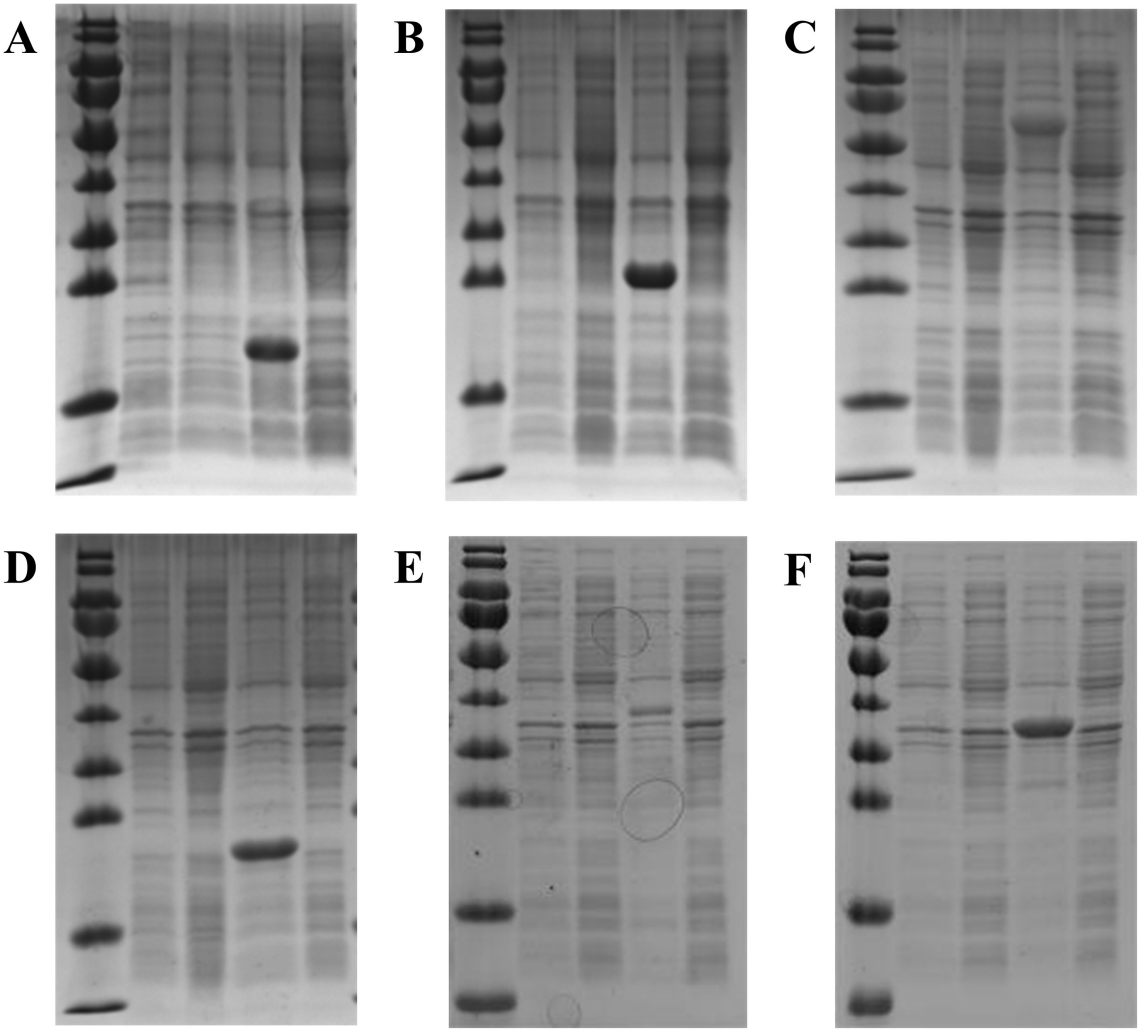
SDS-PAGE of six proteins expressed by recombinant *E. coli* BL21. **(A)** sHsps. **(B)** DPS. **(C)** GroEL. **(D)** USP-1. **(E)** Cu/Zn-SOD. **(F)** USP-2. The lanes (from left to right) correspond to (1) marker, (2) induced control (BL21 PET28a), (3) uninduced control (BL21 PET28a), (4) induced BL21 pET-28a–target protein, and (5) uninduced BL21 pET-28a–target protein.

Six target proteins related to survival at low temperature (according to the transcriptome results of strain RCBS9) were successfully expressed in recombinant *E. coli* BL21. The five SDS-PAGE lanes for these six proteins (from left to right) correspond to (1) marker, (2) induced control (BL21 PET28a), (3) uninduced control (BL21 PET28a), (4) induced BL21 pET-28a–target protein, and (5) uninduced BL21 pET-28a–target protein. The results demonstrated that all six proteins were successfully induced and in the expected positions (Figure 7).

**Figure 8:**
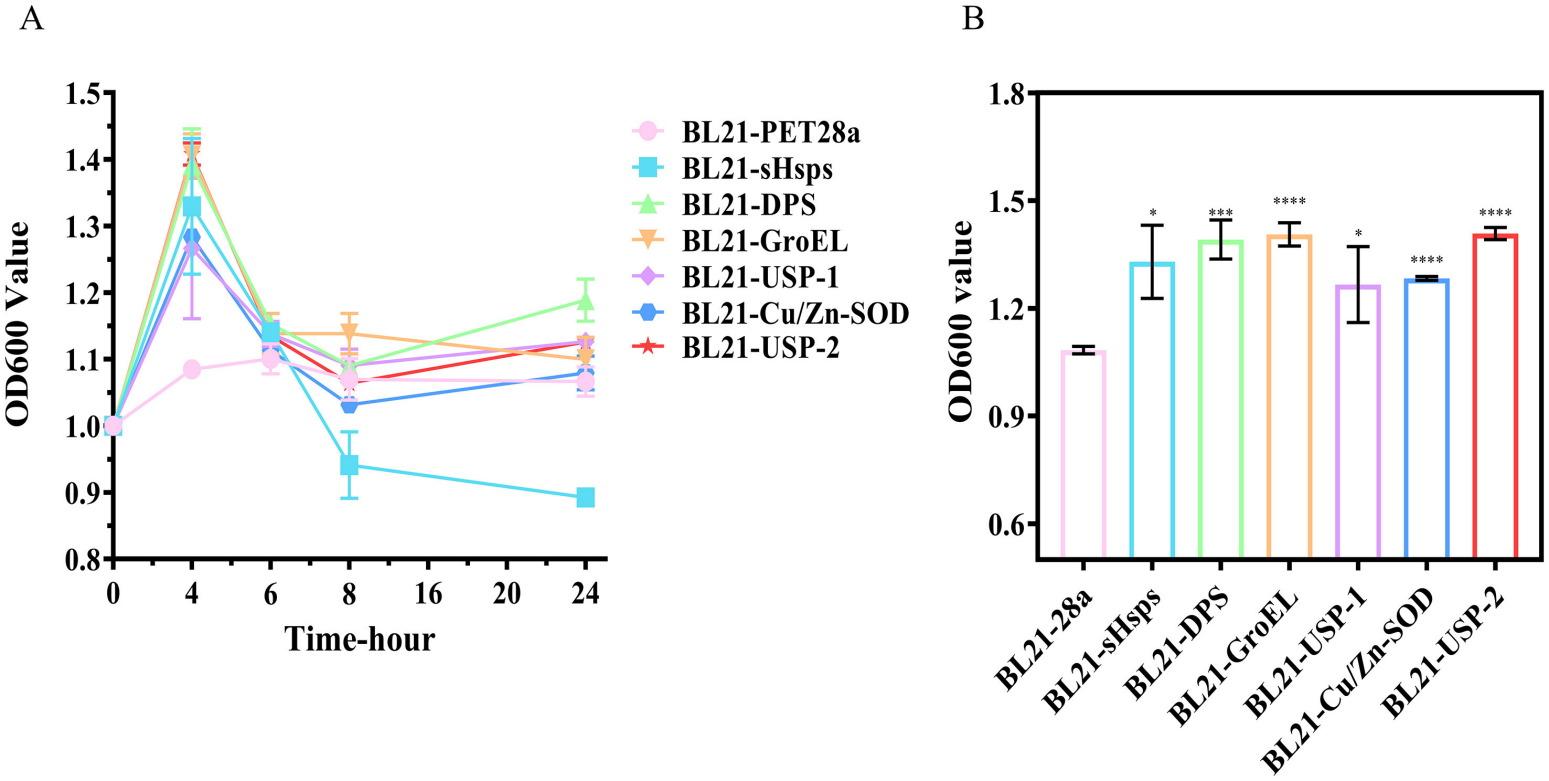
**(A)** Growth curves and **(B)** bacterial load of recombinant *E. coli* at 10°C (4th h, value of bacterial solution at OD600 nm). ns, not statistically different; *P < 0.05, **P < 0.01, ***P < 0.001, ****P < 0.0001.

The growth curves of each recombinant *E. coli* expressing the six target proteins were significantly different from that of the control (BL21-PET28a). The bacterial count of the control (BL21-PET28a) at 10°C was almost unchanged, with a value of about 1-1.1 at OD600 nm. However, the number of recombinant E. coli expressing the target proteins reached the peak at 4 h, and the value at OD600 nm of BL21-DPS, BL21-GroEL, and BL21-USP-2 reached about 1.4, and then began to decline. After 8 h, except for BL21-SHsps, the values of bacterial mass at OD600 nm were around 1.1 for all strains (Figure 8). Based on the results, we speculate that the induced target proteins improve low-temperature adaptation when they exist in large quantities in the recombinant *E. coli*. After 8 h, due to damage and degradation of the proteins, the recombinant *E. coli* could not produce a large amount of target proteins without additional IPTG induction, and the bacterial counts began to decline until they were comparable to those of the control (BL21-PET28a).

In addition to the protective function of the target proteins themselves, the target proteins may produce cascade reactions that increase other protective substances or improve the cellular structure for better survival at low temperature. More in-depth validation analyses of the reasons for the large decrease in the BL21-sHsps bacterial count at 6 h and the regulatory mechanisms underlying the low-temperature tolerance of strain RCBS9 are required. Thereafter, based on the current work, we will further investigate the mechanisms underlying the low-temperature tolerance of strain RCBS9.

## 4 Conclusion

In this experimental study, we found that strain RCBS9 adapts to low temperature in LB medium similarly to other strains. However, in MSM medium, the adaptation strategy of strain RCBS9 diverges under the combined stress of E2 and low temperature. Transcriptomic analysis basically aligned with experimental data, elucidating the gene expression regulatory network of strain RCBS9 under cold stress. Strain RCBS9 up-regulates signal transduction genes (e.g., Desk) and global transcription factors to sustain viability and physiological functions at low temperatures. Notably, strain RCBS9 significantly enhanced fatty acid catabolism for energy production. Unlike common cold shock proteins, strain RCBS9 up-regulates heat shock proteins (HSPs), stress protective protein-USP, and various transporter proteins in response to cold stress. Transcriptome analysis identified six key genes for low-temperature tolerance, whose overexpression in E. coli improved cold adaptation. This work delineates strain RCBS9′s cold adaptation strategy and validates the cryoprotective roles of several genes, providing a theoretical foundation for its application in animal husbandry. Future research should explore strain RCBS9′s adaptation at lower temperatures and further dissect the function and regulation of key cryotolerance genes.

## Data Availability

The original contributions presented in the study are publicly available. This data can be found here: https://www.ncbi.nlm.nih.gov/, accession number PRJNA1156569.

## References

[B1] Adeel M. Song X. Wang Y. Francis D. Yang Y. (2017). Environmental impact of estrogens on human, animal and plant life: a critical review. Environ. Int. 99, 107–119. 10.1016/j.envint.2016.12.010 28040262

[B2] Adeel M. Yang Y. S. Wang Y. Y. Song X. M. Ahmad M. A. Rogers H. J. (2018). Uptake and transformation of steroid estrogens as emerging contaminants influence plant development. Environ. Pollut. 243, 1487–1497. 10.1016/j.envpol.2018.09.016 30292158

[B3] Almeyda M. D. Scodelaro Bilbao P. G. Popovich C. A. Constenla D. Leonardi P. I. (2020). Enhancement of polyunsaturated fatty acid production under low-temperature stress in *Cylindrotheca closterium*. J. Appl. Phycol. 32, 989–1001. 10.1007/s10811-020-02047-x

[B4] Anand A. Chen K. Catoiu E. Sastry A. V. Olson C. A. Sandberg T. E.. (2019). OxyR is a convergent target for mutations acquired during adaptation to oxidative stress-prone metabolic states. Mol. Biol. Evol. 37, 660–667. 10.1093/molbev/msz251 31651953 PMC7038661

[B5] Babu Sait M. R. Koliwer-Brandl H. Stewart J. A. Swarts B. M. Jacobsen M. Ioerger T. R.. (2022). PPE51 mediates uptake of trehalose across the mycomembrane of *Mycobacterium tuberculosis*. Sci. Rep. 12:2097. 10.1038/s41598-022-06109-7 35136132 PMC8826857

[B6] Ballweg S. Sezgin E. Doktorova M. Covino R. Reinhard J. Wunnicke D.. (2020). Regulation of lipid saturation without sensing membrane fluidity. Nat. Commun. 11:756. 10.1038/s41467-020-14528-1 32029718 PMC7005026

[B7] Bao C. Li M. Zhao X. Shi J. Liu Y. Zhang N.. (2023). Mining of key genes for cold adaptation from *Pseudomonas fragi* D12 and analysis of its cold-adaptation mechanism. Front. Microbiol. 14:1215837. 10.3389/fmicb.2023.1215837 37485517 PMC10358777

[B8] Bergmann M. Collard F. Fabres J. Gabrielsen G. W. Provencher J. F. Rochman C. M.. (2022). Plastic pollution in the Arctic. Nat. Rev. Earth Environ. 3, 323–337. 10.1038/s43017-022-00279-8

[B9] Borisov V. B. Siletsky S. A. Nastasi M. R. Forte E. (2021). ROS defense systems and terminal oxidases in bacteria. Antioxidants 10:839. 10.3390/antiox10060839 34073980 PMC8225038

[B10] Bremer E. Krämer R. (2019). Responses of microorganisms to osmotic stress. Ann. Rev. Microbiol. 73, 313–334. 10.1146/annurev-micro-020518-115504 31180805

[B11] Camba C. Walter-Lakes B. Digal P. Taheri-Araghi S. Bezryadina A. (2024). Biofilm formation and manipulation with optical tweezers. Biomed. Opt. Expr. 15, 1181–1191. 10.1364/BOE.510836 38404331 PMC10890877

[B12] Cava F. de Pedro M. A. Lam H. Davis B. M. Waldor M. K. (2011). Distinct pathways for modification of the bacterial cell wall by non-canonical D-amino acids. EMBO J. 30, 3442–3453. 10.1038/emboj.2011.246 21792174 PMC3160665

[B13] Chainy G. B. N. Sahoo D. K. (2020). Hormones and oxidative stress: an overview. Free Rad. Res. 54, 1–26. 10.1080/10715762.2019.1702656 31868060

[B14] Chembazhi U. V. Patil V. V. Sah S. Reeve W. Tiwari R. P. Woo E.. (2017). Uracil DNA glycosylase (UDG) activities in *Bradyrhizobium diazoefficiens*: characterization of a new class of UDG with broad substrate specificity. Nucl. Acids Res. 45, 5863–5876. 10.1093/nar/gkx209 28369586 PMC5449639

[B15] Chen L. Ma Y. Ma X. Liu L. Jv X. Li A.. (2023). TFEB regulates cellular labile iron and prevents ferroptosis in a TfR1-dependent manner. Free Rad. Biol. Med. 208, 445–457. 10.1016/j.freeradbiomed.2023.09.004 37683766

[B16] Chen Y. Liu H. Zhang H. Sun C. Hu Z. Tian Q.. (2016). And-1 coordinates with CtIP for efficient homologous recombination and DNA damage checkpoint maintenance. Nucl. Acids Res. 45, 2516–2530. 10.1093/nar/gkw1212 27940552 PMC5389581

[B17] Chia G. W. N. Seviour T. Kjelleberg S. Hinks J. (2021). Carotenoids improve bacterial tolerance towards biobutanol through membrane stabilization. Environ. Sci. 8, 328–341. 10.1039/D0EN00983K

[B18] Ciślak M. Kruszelnicka I. Zembrzuska J. Ginter-Kramarczyk D. (2023). Estrogen pollution of the European aquatic environment: a critical review. Water Res. 229:119413. 10.1016/j.watres.2022.119413 36470046

[B19] Culham D. E. Marom D. Boutin R. Garner J. Ozturk T. N. Sahtout N.. (2018). Dual role of the C-terminal domain in osmosensing by bacterial osmolyte transporter ProP. Biophys. J. 115, 2152–2166. 10.1016/j.bpj.2018.10.023 30448037 PMC6289098

[B20] Curtis N. A. C. Hughes J. M. Ross G. W. (1976). Inhibition of peptidoglycan cross-linking in growing cells of *Escherichia coli* by penicillins and cephalosporins, and its prevention by R factor-mediated beta-lactamase. Antimicrob. Agents Chemother. 9, 208–213. 10.1128/AAC.9.2.208 773294 PMC429505

[B21] Cybulski L. E. Martín M. Mansilla M. C. Fernández A. de Mendoza D. (2010). Membrane thickness cue for cold sensing in a bacterium. Curr. Biol. 20, 1539–1544. 10.1016/j.cub.2010.06.074 20705470

[B22] Deng L.-Q. Yu H.-Q. Liu Y.-P. Jiao P.-P. Zhou S.-F. Zhang S.-Z.. (2014). Heterologous expression of antifreeze protein gene AnAFP from *Ammopiptanthus nanus* enhances cold tolerance in *Escherichia coli* and tobacco. Gene 539, 132–140. 10.1016/j.gene.2014.01.013 24502990

[B23] Devi K. P. Sakthivel R. Nisha S. A. Suganthy N. Pandian S. K. (2013). Eugenol alters the integrity of cell membrane and acts against the nosocomial pathogen *Proteus mirabilis*. Archiv. Pharm. Res. 36, 282–292. 10.1007/s12272-013-0028-3 23444040

[B24] Dik D. A. Zhang N. Sturgell E. J. Sanchez B. B. Chen J. S. Webb B.. (2021). A synthetic 5,3-cross-link in the cell wall of rod-shaped Gram-positive bacteria. Proc. Natl. Acad. Sci. U. S. A. 118:e2100137118. 10.1073/pnas.2100137118 33836615 PMC7980369

[B25] Druger-Liotta J. Prange V. J. Overdier D. G. Csonka L. N. (1987). Selection of mutations that alter the osmotic control of transcription of the *Salmonella typhimurium* proU operon. J. Bacteriol. 169, 2449–2459. 10.1128/jb.169.6.2449-2459.1987 3294791 PMC212089

[B26] Du B. Wang S. Chen G. Wang G. Liu L. (2022). Nutrient starvation intensifies chlorine disinfection-stressed biofilm formation. Chemosphere 295:133827. 10.1016/j.chemosphere.2022.133827 35122818

[B27] Dubrez L. Causse S. Borges Bonan N. Dumétier B. Garrido C. (2020). Heat-shock proteins: chaperoning DNA repair. Oncogene 39, 516–529. 10.1038/s41388-019-1016-y, 31541194

[B28] E J. Chen J. Chen Z. Ma R. Zhang J. Yao C.. (2021). Effects of different initial pH values on freeze-drying resistance of *Lactiplantibacillus plantarum* LIP-1 based on transcriptomics and proteomics. Food Res. Int. 149:110694. 10.1016/j.foodres.2021.110694 34600689

[B29] Fang Q.-j. Han Y.-x. Shi Y.-j. Huang H.-q. Fang Z.-g. Hu Y.-h. (2019). Universal stress proteins contribute *Edwardsiella piscicida* adversity resistance and pathogenicity and promote blocking host immune response. Fish Shellf. Immunol. 95, 248–258. 10.1016/j.fsi.2019.10.035 31654767

[B30] Flores N. Hoyos S. Venegas M. Galetovi,ć A. Zúñiga L. M. Fábrega F.. (2020). *Haloterrigena* sp. strain SGH1, a bacterioruberin-rich, perchlorate-tolerant halophilic archaeon isolated from halite microbial communities, Atacama Desert, Chile. Front. Microbiol. 11:324. 10.3389/fmicb.2020.00324 32194531 PMC7066086

[B31] Fornelos N. Browning D. F. Butala M. (2016). The use and abuse of LexA by mobile genetic elements. Trends Microbiol. 24, 391–401. 10.1016/j.tim.2016.02.009 26970840

[B32] Galhardo R. S. Do R. Yamada M. Friedberg E. C. Hastings P. J. Nohmi T.. (2009). DinB upregulation is the sole role of the SOS response in stress-induced mutagenesis in *Escherichia coli*. Genetics 182, 55–68. 10.1534/genetics.109.100735 19270270 PMC2674841

[B33] Gao B. Liang L. Su L. Wen A. Zhou C. Feng Y. (2023). Structural basis for regulation of SOS response in bacteria. Proc. Natl. Acad. Sci. U. S. A. 120:e2217493120. 10.1073/pnas.2217493120 36598938 PMC9926225

[B34] Gao X. Li P. Mei J. Xie J. (2021). TMT-based quantitative proteomics analysis of the fish-borne spoiler *Shewanella putrefaciens* subjected to cold stress using LC-MS/MS. J. Chem. 2021:8876986. 10.1155/2021/8876986

[B35] Gao Y. Liu Y. Liu Y. Peng Y. Yuan B. Fu Y.. (2021). UHRF1 promotes androgen receptor-regulated CDC6 transcription and anti-androgen receptor drug resistance in prostate cancer through KDM4C-mediated chromatin modifications. Cancer Lett. 520, 172–183. 10.1016/j.canlet.2021.07.012 34265399

[B36] Garde S. Chodisetti P. K. Reddy M. (2021). Peptidoglycan: structure, synthesis, and regulation. EcoSal Plus 9:2020. 10.1128/ecosalplus.ESP-0010-2020 33470191 PMC11168573

[B37] Ghaedamini H. Duanghathaipornsuk S. Onusko P. Binsheheween A. M. Kim D.-S. (2023). Reduced glutathione-modified electrode for the detection of hydroxyl free radicals. Biosensors 13:254. 10.3390/bios13020254 36832020 PMC9953857

[B38] Goddard A. D. Bali S. Mavridou D. A. I. Luque-Almagro V. M. Gates A. J. Dolores Roldán M.. (2017). The *Paracoccus denitrificans* NarK-like nitrate and nitrite transporters-probing nitrate uptake and nitrate/nitrite exchange mechanisms. Mol. Microbiol. 103, 117–133. 10.1111/mmi.13546 27696579 PMC5217062

[B39] Goltermann L. Sarusie M. V. Bentin T. (2016). Chaperonin GroEL/GroES over-expression promotes aminoglycoside resistance and reduces drug susceptibilities in *Escherichia coli* following exposure to sublethal aminoglycoside doses. Front. Microbiol. 6:1572. 10.3389/fmicb.2015.01572 26858694 PMC4726795

[B40] Gregson B. H. Metodieva G. Metodiev M. V. Golyshin P. N. McKew B. A. (2020). Protein expression in the obligate hydrocarbon-degrading psychrophile *Oleispira antarctica* RB-8 during alkane degradation and cold tolerance. Environ. Microbiol. 22, 1870–1883. 10.1111/1462-2920.14956 32090431 PMC7318663

[B41] Han M. Zhang Z. Liu S. Sheng Y. Waigi M. G. Hu X.. (2023). Genotoxicity of organic contaminants in the soil: a review based on bibliometric analysis and methodological progress. Chemosphere 313:137318. 10.1016/j.chemosphere.2022.137318 36410525

[B42] Hao P. Lv Z. Pan H. Zhang J. Wang L. Zhu Y.. (2024). Characterization and low-temperature biodegradation mechanism of 17β-estradiol-degrading bacterial strain *Rhodococcus* sp. RCBS9. Environ. Res. 240:117513. 10.1016/j.envres.2023.117513 37890824

[B43] Hao P. Lv Z. Wu S. Zhang X. Gou C. Wang L.. (2023). Transcriptome profiling of *Microbacterium resistens* MZT7 reveals mechanisms of 17β-estradiol response and biotransformation. Environ. Res. 217:114963. 10.1016/j.envres.2022.114963 36471558

[B44] Herndon J. L. Peters R. E. Hofer R. N. Simmons T. B. Symes S. J. Giles D. K. (2020). Exogenous polyunsaturated fatty acids (PUFAs) promote changes in growth, phospholipid composition, membrane permeability and virulence phenotypes in *Escherichia coli*. BMC Microbiol. 20:305. 10.1186/s12866-020-01988-0 33046008 PMC7552566

[B45] Hoskisson P. A. Hutchings M. I. (2006). MtrAB-LpqB: a conserved three-component system in actinobacteria? Trends Microbiol. 14, 444–449. 10.1016/j.tim.2006.08.005 16934981

[B46] Husain A. A. Pinto S. M. Agarwal N. Behera S. K. Khulkhule P. R. Bhartiya N. M.. (2022). Comprehensive proteomic analysis of *Brucella melitensis* ATCC23457 strain reveals metabolic adaptations in response to nutrient stress. Curr. Microbiol. 80:20. 10.1007/s00284-022-03105-y 36460801

[B47] Inda M. E. Vazquez D. B. Fernández A. Cybulski L. E. (2019). Reverse engineering of a thermosensing regulator switch. J. Mol. Biol. 431, 1016–1024. 10.1016/j.jmb.2019.01.025 30738600

[B48] Irshad A. Rehman R. N. U. Kareem H. A. Yang P. Hu T. (2021). Addressing the challenge of cold stress resilience with the synergistic effect of rhizobium inoculation and exogenous melatonin application in *Medicago truncatula*. Ecotoxicol. Environ. Saf. 226:112816. 10.1016/j.ecoenv.2021.112816 34597844

[B49] Jin S. Wang Y. Zhao X. (2022). Cold-adaptive mechanism of psychrophilic bacteria in food and its application. Microb. Pathog. 169:105652. 10.1016/j.micpath.2022.105652 35753601

[B50] Kar A. Deole S. Nayak R. R. Gupta A. K. Gadratagi B. G. Patil N.. (2024). Distribution and risk assessment of pesticide pollution in small streams adjoining paddy fields. J. Hazard. Mater. 469:133852. 10.1016/j.jhazmat.2024.133852 38430593

[B51] Kizawa A. Kawahara A. Takashima K. Takimura Y. Nishiyama Y. Hihara Y. (2017). The LexA transcription factor regulates fatty acid biosynthetic genes in the cyanobacterium *Synechocystis* sp. PCC 6803. Plant J. 92, 189–198. 10.1111/tpj.13644 28744961

[B52] Kloska A. Cech G. M. Sadowska M. Krause K. Szalewska-Pałasz A. Olszewski P. (2020). Adaptation of the marine bacterium *Shewanella baltica* to low temperature stress. Int. J. Mol. Sci. 21:124338. 10.3390/ijms21124338 32570789 PMC7352654

[B53] Kong Y. Yan H. Hu J. Dang Y. Han Z. Tian B.. (2024). Antibacterial activity and mechanism of action of osthole against *Listeria monocytogenes*. J. Agricult. Food Chem. 72, 10853–10861. 10.1021/acs.jafc.3c07931 38708871

[B54] Körner H. Sofia H. J. Zumft W. G. (2003). Phylogeny of the bacterial superfamily of Crp-Fnr transcription regulators: exploiting the metabolic spectrum by controlling alternative gene programs. FEMS Microbiol. Rev. 27, 559–592. 10.1016/S0168-6445(03)00066-4 14638413

[B55] Krol E. Werel L. Essen L. O. Becker A. (2023). Structural and functional diversity of bacterial cyclic nucleotide perception by CRP proteins. microLife 4:uqad024. 10.1093/femsml/uqad024 37223727 PMC10187061

[B56] Kuttel M. M. Cescutti P. Distefano M. Rizzo R. (2017). Fluorescence and NMR spectroscopy together with molecular simulations reveal amphiphilic characteristics of a Burkholderia biofilm exopolysaccharide. J. Biol. Chem. 292, 11034–11042. 10.1074/jbc.M117.785048 28468829 PMC5491786

[B57] Li C. Murugaiyan J. Thomas C. Alter T. Riedel C. (2020). Isolate specific cold response of *Yersinia enterocolitica* in transcriptional, proteomic, and membrane physiological changes. Front. Microbiol. 10:3037. 10.3389/fmicb.2019.03037 32038527 PMC6990146

[B58] Li C. Xu Y. Li Z. Cheng P. Yu G. (2022). Transcriptomic and metabolomic analysis reveals the potential mechanisms underlying the improvement of β-carotene and torulene production in *Rhodosporidiobolus colostri* under low temperature treatment. Food Res. Int. 156:111158. 10.1016/j.foodres.2022.111158 35651024

[B59] Li R. Yang Y. Cao H. Peng X. Yu Q. He L.. (2023). Heterologous expression of the tobacco metallothionein gene NtMT2F confers enhanced tolerance to Cd stress in *Escherichia coli* and *Arabidopsis thaliana*. Plant Physiol. Biochem. 195, 247–255. 10.1016/j.plaphy.2023.01.027 36645929

[B60] Li Y.-F. Lin Y.-T. Wang Y.-Q. Ni J.-Y. Power D. M. (2023). Ioxynil and diethylstilbestrol impair cardiac performance and shell growth in the mussel *Mytilus coruscus*. Sci. Tot. Environ. 905:166834. 10.1016/j.scitotenv.2023.166834 37717744

[B61] Licht A. Bommer M. Werther T. Neumann K. Hobe C. Schneider E. (2019). Structural and functional characterization of a maltose/maltodextrin ABC transporter comprising a single solute binding domain (MalE) fused to the transmembrane subunit MalF. Res. Microbiol. 170, 1–12. 10.1016/j.resmic.2018.08.006 30193862

[B62] Lin Y.-T. Wang Y.-C. Xue Y.-M. Tong Z. Jiang G.-Y. Hu X.-R.. (2024). Decoding the influence of low temperature on biofilm development: the hidden roles of c-di-GMP. Sci. Tot. Environ. 927:172376. 10.1016/j.scitotenv.2024.172376 38604376

[B63] Liu H. Zhang C. Zhang X. Tan K. Zhang H. Cheng D.. (2020). A novel carotenoids-producing marine bacterium from noble scallop *Chlamys nobilis* and antioxidant activities of its carotenoid compositions. Food Chem. 320:126629. 10.1016/j.foodchem.2020.126629 32203829

[B64] Lv X. Cheng J.-H. (2022). Evaluation of the effects of cold plasma on cell membrane lipids and oxidative injury of *Salmonella typhimurium*. Molecules 27:640. 10.3390/molecules27030640 35163904 PMC8838372

[B65] Marchenkov V. Gorokhovatsky A. Marchenko N. Ivashina T. Semisotnov G. (2020). Back to GroEL-assisted protein folding: GroES binding-induced displacement of denatured proteins from GroEL to bulk solution. Biomolecules 10:162. 10.3390/biom10010162 31968530 PMC7022901

[B66] McGroty S. E. Pattaniyil D. T. Patin D. Blanot D. Ravichandran A. C. Suzuki H.. (2013). Biochemical characterization of UDP-N-acetylmuramoyl-L-alanyl-D-glutamate: meso-2,6-diaminopimelate ligase (MurE) from *Verrucomicrobium spinosum* DSM 4136T. PLoS ONE 8:e66458. 10.1371/journal.pone.0066458 23785498 PMC3681970

[B67] Méndez V. Rodríguez-Castro L. Durán R. E. Padrón G. Seeger M. (2022). The OxyR and SoxR transcriptional regulators are involved in a broad oxidative stress response in *Paraburkholderia xenovorans* LB400. Biol. Res. 55:7. 10.1186/s40659-022-00373-7 35184754 PMC8859910

[B68] Miyamoto T. Homma H. (2021). D-Amino acid metabolism in bacteria. J. Biochem. 170, 5–13. 10.1093/jb/mvab043 33788945

[B69] Park E.-J. Kwon Y.-M. Lee J.-W. Kang H.-Y. Oh J.-I. (2019). Dual control of RegX3 transcriptional activity by SenX3 and PknB. J. Biol. Chem. 294, 11023–11034. 10.1074/jbc.RA119.008232 31160336 PMC6635444

[B70] Pátek M. Grulich M. Nešvera J. (2021). Stress response in Rhodococcus strains. Biotechnol. Adv. 53:107698. 10.1016/j.biotechadv.2021.107698 33515672

[B71] Perdih A. Hodoscek M. Solmajer T. (2009). MurD ligase from *E. coli*: tetrahedral intermediate formation study by hybrid quantum mechanical/molecular mechanical replica path method. Proteins 74, 744–759. 10.1002/prot.22188 18704940

[B72] Rath H. Reder A. Hoffmann T. Hammer E. Seubert A. Bremer E.. (2020). Management of osmoprotectant uptake hierarchy in *Bacillus subtilis* via a SigB-dependent antisense RNA. Front. Microbiol. 11:622. 10.3389/fmicb.2020.00622 32373088 PMC7186363

[B73] Ristovski M. Farhat D. Bancud S. E. M. Lee J.-Y. (2021). Lipid transporters beam signals from cell membranes. Membranes 11:562. 10.3390/membranes11080562 34436325 PMC8399137

[B74] Rosenzweig R. Nillegoda N. B. Mayer M. P. Bukau B. (2019). The Hsp70 chaperone network. Nat. Rev. Mol. Cell Biol. 20, 665–680. 10.1038/s41580-019-0133-3 31253954

[B75] Sacdal R. Madriaga J. Espino M. P. (2020). Overview of the analysis, occurrence and ecological effects of hormones in lake waters in Asia. Environ. Res. 182:109091. 10.1016/j.envres.2019.109091 31927242

[B76] Salla R. F. Costa M. J. Abdalla F. C. Oliveira C. R. Tsukada E. Boeing G. A. N. S.. (2024). Estrogen contamination increases vulnerability of amphibians to the deadly chytrid fungus. Sci. Tot. Environ. 917:170337. 10.1016/j.scitotenv.2024.170337 38301782

[B77] Salton M. R. J. (1967). Structure and function of bacterial cell membranes. Ann. Rev. Microbiol. 21, 417–442. 10.1146/annurev.mi.21.100167.002221 4860264

[B78] Shahu S. Vtyurina N. Das M. Meyer A. S. Ganji M. Abbondanzieri E. A. (2024). Bridging DNA contacts allow Dps from *E. coli* to condense DNA. Nucl. Acids Res. 52, 4456–4465. 10.1093/nar/gkae223 38572752 PMC11077075

[B79] Shan C. Wu H. Zhou J. Yan W. Zhang J. Liu X. (2020). Synergistic effects of bacteriocin from *Lactobacillus panis* C-M2 combined with dielectric barrier discharged non-thermal plasma (DBD-NTP) on *Morganella* sp. in Aquatic Foods. Antibiotics 9:593. 10.3390/antibiotics9090593 32927848 PMC7557774

[B80] Shi G. Kim H. Koo S. (2022). Oxo-carotenoids as efficient superoxide radical scavengers. Antioxidants 11:81525. 10.3390/antiox11081525 36009244 PMC9405038

[B81] Sleator R. D. Hill C. (2002). Bacterial osmoadaptation: the role of osmolytes in bacterial stress and virulence. FEMS Microbiol. Rev. 26, 49–71. 10.1111/j.1574-6976.2002.tb00598.x 12007642

[B82] Smith M. R. Fernandes J. Go Y.-M. Jones D. P. (2017). Redox dynamics of manganese as a mitochondrial life-death switch. Biochem. Biophys. Res. Commun. 482, 388–398. 10.1016/j.bbrc.2016.10.126 28212723 PMC5382988

[B83] Song Q. Li X. Hou N. Pei C. Li D. (2024). Chemotaxis-mediated degradation of PAHs and heterocyclic PAHs under low-temperature stress by *Pseudomonas fluorescens* S01: insights into the mechanisms of biodegradation and cold adaptation. J. Hazard. Mater. 469:133905. 10.1016/j.jhazmat.2024.133905 38422734

[B84] Song W.-S. Kim S.-M. Jo S.-H. Lee J.-S. Jeon H.-J. Ko B. J.. (2020). Multi-omics characterization of the osmotic stress resistance and protease activities of the halophilic bacterium *Pseudoalteromonas phenolica* in response to salt stress. RSC Adv. 10, 23792–23800. 10.1039/D0RA04034G 35517354 PMC9054934

[B85] Song Y. Deng S. P. Acosta-Martínez V. Katsalirou E. (2008). Characterization of redox-related soil microbial communities along a river floodplain continuum by fatty acid methyl ester (FAME) and 16S rRNA genes. Appl. Soil Ecol. 40, 499–509. 10.1016/j.apsoil.2008.07.005

[B86] Stahl M. Korotkov V. S. Balogh D. Kick L. M. Gersch M. Pahl A.. (2018). Selective activation of human caseinolytic protease P (ClpP). Angewandte Chemie Int. Ed 57, 14602–14607. 10.1002/anie.201808189 30129683

[B87] Stautz J. Hellmich Y. Fuss M. F. Silberberg J. M. Devlin J. R. Stockbridge R. B.. (2021). Molecular mechanisms for bacterial potassium homeostasis. J. Mol. Biol. 433:166968. 10.1016/j.jmb.2021.166968 33798529 PMC9041122

[B88] Sun S. Abdellah Y. A. Y. Miao L. Wu B. Ma T. Wang Y.. (2022). Impact of microbial inoculants combined with humic acid on the fate of estrogens during pig manure composting under low-temperature conditions. J. Hazard. Mater. 424:127713. 10.1016/j.jhazmat.2021.127713 34815123

[B89] Tribelli P. M. López N. I. (2011). Poly(3-hydroxybutyrate) influences biofilm formation and motility in the novel Antarctic species *Pseudomonas extremaustralis* under cold conditions. Extremophiles 15:541. 10.1007/s00792-011-0384-1 21667094

[B90] Unnikrishan A. Khalid N. K. Rayaroth M. P. Thomas S. Nazim A. Aravindakumar C. T.. (2024). Occurrence and distribution of steroid hormones (estrogen) and other contaminants of emerging concern in a south indian water body. Chemosphere 351:141124. 10.1016/j.chemosphere.2024.141124 38211796

[B91] Velasco L. Dublang L. Moro F. Muga A. (2019). The complex phosphorylation patterns that regulate the activity of Hsp70 and its cochaperones. Int. J. Mol. Sci. 20:4122. 10.3390/ijms20174122 31450862 PMC6747476

[B92] Wang C. Chen Y. Zhou H. Li X. Tan Z. (2020). Adaptation mechanisms of *Rhodococcus* sp. CNS16 under different temperature gradients: physiological and transcriptome. Chemosphere 238:124571. 10.1016/j.chemosphere.2019.124571 31472351

[B93] Wang L. Chakravarthy S. Verdine G. L. (2017). Structural basis for the lesion-scanning mechanism of the MutY DNA glycosylase. J. Biol. Chem. 292, 5007–5017. 10.1074/jbc.M116.757039 28130451 PMC5377813

[B94] Wang R. C. Wen X. H. Qian Y. (2006). Microbial population structure changes in a suspended carrier biofilm reactor. Water Sci. Technol. 54, 145–153. 10.2166/wst.2006.740 17167898

[B95] Wang Y. Chen X. Wu B. Ma T. Jiang H. Mi Y.. (2022a). Potential and mechanism for bioremediation of papermaking black liquor by a psychrotrophic lignin-degrading bacterium, *Arthrobacter* sp. C2. J. Hazard. Mater. 439:129534. 10.1016/j.jhazmat.2022.129534 35850064

[B96] Wang Y. Huang Z. Zhou N. Liu C. Jiang C. Li D.. (2022b). The response regulator FlmD regulates biofilm formation in *Comamonas testosteroni* through the transcriptional activator SoxR. Microorganisms 10:356. 10.3390/microorganisms10020356 35208812 PMC8880074

[B97] Wang Y. Wang F. Zhang X. Cen C. Fu L. (2020). Transcription factors FabR and FadR regulate cold adaptability and spoilage potential of *Shewanella baltica*. Int. J. Food Microbiol. 331:108693. 10.1016/j.ijfoodmicro.2020.108693 32535524

[B98] Wang Y. Wu Y. Niu H. Liu Y. Ma Y. Wang X.. (2023). Different cellular fatty acid pattern and gene expression of planktonic and biofilm state *Listeria monocytogenes* under nutritional stress. Food Res. Int. 167:112698. 10.1016/j.foodres.2023.112698 37087265

[B99] Wiesmann C. L. Zhang Y. Alford M. Hamilton C. D. Dosanjh M. Thoms D.. (2023). The ColR/S two-component system is a conserved determinant of host association across *Pseudomonas* species. ISME J. 17, 286–296. 10.1038/s41396-022-01343-3 36424517 PMC9859794

[B100] Xikeranmu Z. Ma J. Liu X. (2020). Characterization of a Mn-SOD from the desert beetle *Microdera punctipennis* and its increased resistance to cold stress in *E. coli* cells. PeerJ 8:e8507. 10.7717/peerj.8507 32095349 PMC7025704

[B101] Xu K. Lin L. Shen D. Chou S.-H. Qian G. (2021). Clp is a “busy” transcription factor in the bacterial warrior, *Lysobacter enzymogenes*. Comput. Struct. Biotechnol. J. 19, 3564–3572. 10.1016/j.csbj.2021.06.020 34257836 PMC8246147

[B102] Yakimov A. Pobegalov G. Bakhlanova I. Khodorkovskii M. Petukhov M. Baitin D. (2017). Blocking the RecA activity and SOS-response in bacteria with a short α-helical peptide. Nucl. Acids Res. 45, 9788–9796. 10.1093/nar/gkx687 28934502 PMC5766188

[B103] Yan J. Xie J. (2020). Comparative proteome analysis of *Shewanella putrefaciens* WS13 mature biofilm under cold stress. Front. Microbiol. 11:1225. 10.3389/fmicb.2020.01225 32582122 PMC7296144

[B104] Yan T. Li M. Wang Q. Wang M. Liu L. Ma C.. (2024). Structures, functions, and regulatory networks of universal stress proteins in clinically relevant pathogenic Bacteria. Cell. Signal. 116:111032. 10.1016/j.cellsig.2023.111032 38185228

[B105] Yang S.-P. Xie J. Cheng Y. Zhang Z. Zhao Y. Qian Y.-F. (2020). Response of *Shewanella putrefaciens* to low temperature regulated by membrane fluidity and fatty acid metabolism. LWT 117:108638. 10.1016/j.lwt.2019.108638

[B106] Yang W. Li J. Yao Z. Li M. (2024). A review on the alternatives to antibiotics and the treatment of antibiotic pollution: current development and future prospects. Sci. Tot. Environ. 926:171757. 10.1016/j.scitotenv.2024.171757 38513856

[B107] Yang Y. Park S.-H. Alford-Zappala M. Lee H.-W. Li J. Cunningham R. P.. (2019). Role of endonuclease III enzymes in uracil repair. Mut. Res. Fund. Mol. Mech. Mutag. 813, 20–30. 10.1016/j.mrfmmm.2018.12.001 30590231 PMC6378108

[B108] Yu M. Jiang C. Meng Y. Wang F. Qian J. Fei F.. (2023). Effect of low temperature on the resistance of Listeria monocytogenes and *Escherichia coli* O157:H7 to acid electrolyzed water. Food Res. Int. 168:112776. 10.1016/j.foodres.2023.112776 37120223

[B109] Yu Z. Wang R. Zhang T. Wang T. Nwanade C. F. Pei T.. (2023). The genome-wide characterization and associated cold-tolerance function of the superoxide dismutase in the cold response of the tick *Haemaphysalis longicornis*. Pesticide Biochem. Physiol. 195:105573. 10.1016/j.pestbp.2023.105573 37666626

[B110] Zhang D. Li S. H.-J. King C. G. Wingreen N. S. Gitai Z. Li Z. (2022). Global and gene-specific translational regulation in *Escherichia coli* across different conditions. PLoS Comput. Biol. 18:e1010641. 10.1371/journal.pcbi.1010641 36264977 PMC9624429

[B111] Zhang L. Yang L. Dong T. Yang J. Dou Q. Ni S.-Q.. (2024). Response of anammox consortia to inhibition from high ferroferric oxide nanoparticles concentration and potential recovery mechanism. Bioresour. Technol. 402:130808. 10.1016/j.biortech.2024.130808 38723724

[B112] Zhang M. Yao M. Lai T. Zhao H. Wang Y. Yang Z. (2021). Response of *Lactiplantibacillus plantarum* NMGL2 to combinational cold and acid stresses during storage of fermented milk as analyzed by data-independent acquisition proteomics. Foods 10:71514. 10.3390/foods10071514 34209263 PMC8305577

[B113] Zhao H. Zhang R. Yan X. Fan K. (2021). Superoxide dismutase nanozymes: an emerging star for anti-oxidation. J. Mater. Chem. B. 9, 6939–6957. 10.1039/D1TB00720C 34161407

[B114] Zhao N.-L. Zhu Z.-Q. Feng H.-Z. Song Y.-J. Huang Q. Mou X.-Y.. (2023). Host-derived peptide signals regulate *Pseudomonas aeruginosa* virulence stress via the ParRS and CprRS two-component systems. J. Hazard. Mater. 460:132512. 10.1016/j.jhazmat.2023.132512 37703740

[B115] Zhao Y. Lv B. Sun F. Liu J. Wang Y. Gao Y.. (2020). Rapid freezing enables aminoglycosides to eradicate bacterial persisters via enhancing mechanosensitive channel MscL-mediated antibiotic uptake. mBio 11:3219. 10.1128/mBio.03239-19 32047133 PMC7018644

[B116] Zhou Y. Li Q. Pan R. Wang Q. Zhu X. Yuan C.. (2022). Regulatory roles of three miRNAs on allergen mRNA expression in *Tyrophagus putrescentiae*. Allergy. 77, 469–482. 10.1111/all.15111 34570913

[B117] Zhu T. Wang W. Wang H. Zhao Y. Qu D. Wu Y. (2022). Mutation of gdpS gene induces a viable but non-culturable state in *Staphylococcus epidermidis* and changes in the global transcriptional profile. BMC Microbiol. 22:288. 10.1186/s12866-022-02708-6 36457079 PMC9714401

[B118] Zou S. Cheng J.-H. (2024). Insights into antioxidative metabolic mechanism of the sub-lethal injured *Listeria monocytogenes* stressed by cold plasma active species for precise control. Food Control. 157:110197. 10.1016/j.foodcont.2023.110197

