# Supplementary material for: Mechanisms underlying the low-temperature adaptation of 17β-estradiol-degrading bacterial strain Rhodococcus sp. RCBS9: insights from physiological and transcriptomic analyses
Source: Front Microbiol. 2024 Nov 21;15:1465627. doi: 10.3389/fmicb.2024.1465627 (PMC11617531; doi:10.3389/fmicb.2024.1465627)
Supplement: Supplementary file 1 [file Data_Sheet_1.docx]

Supplementary Material

# Supplementary Figures and Tables

## Supplementary Figures

**
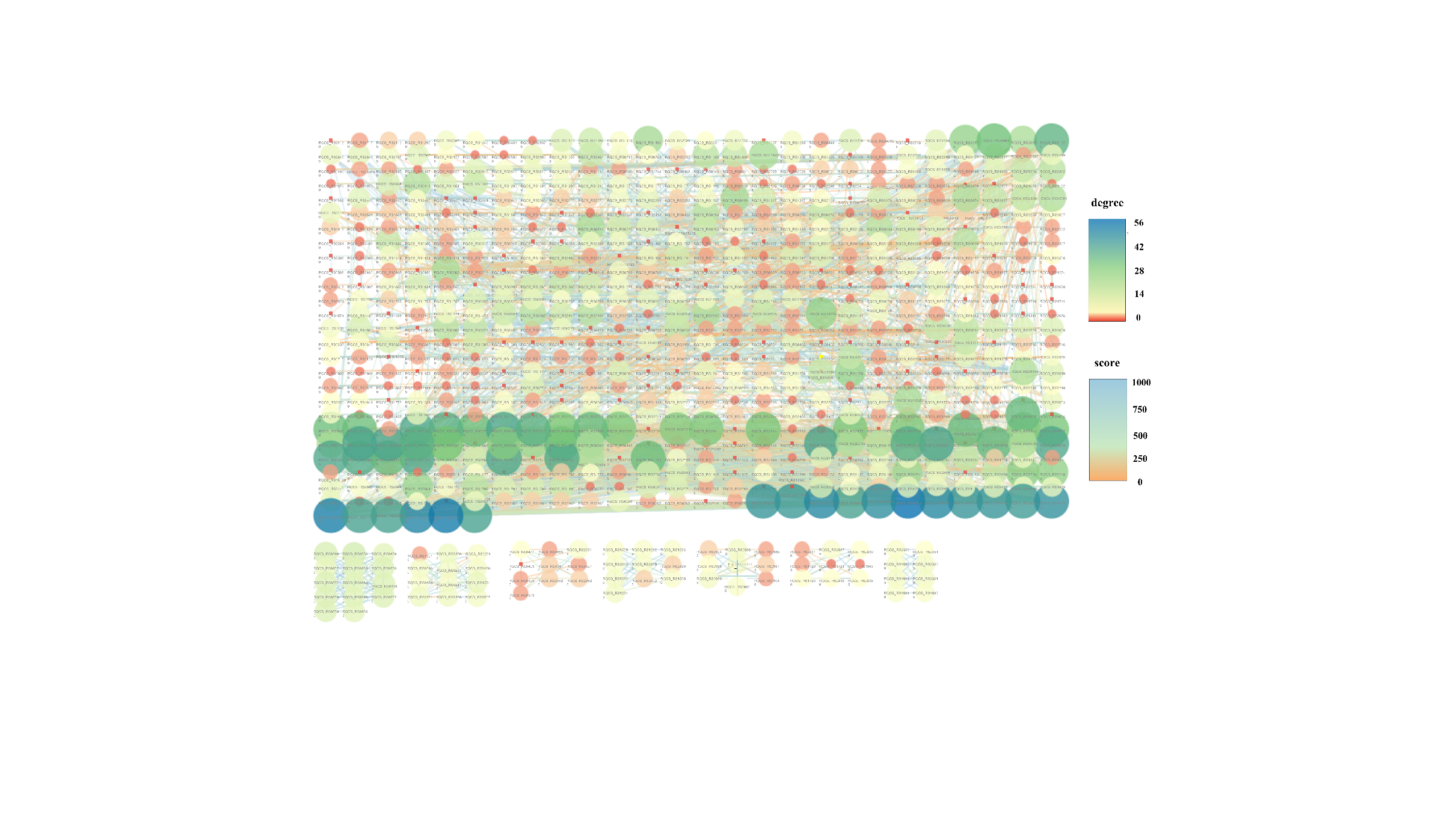
**

**Supplementary Figure 1.** Protein-protein interaction (PPI) network analysis centred on proteins that upregulate differentially expressed genes obtained from transcriptome analysis. The colour gradient from blue to orange symbolises the node degree - the number of proteins that directly interact with a particular protein. The higher the node degree, which tends to be blue, the larger the size of the node. The lines connecting the nodes are colour-coded according to the interaction score (a quantitative metric that assesses the probability or strength of an interaction between two proteins). The higher the interaction score, the bluer it tends to be.


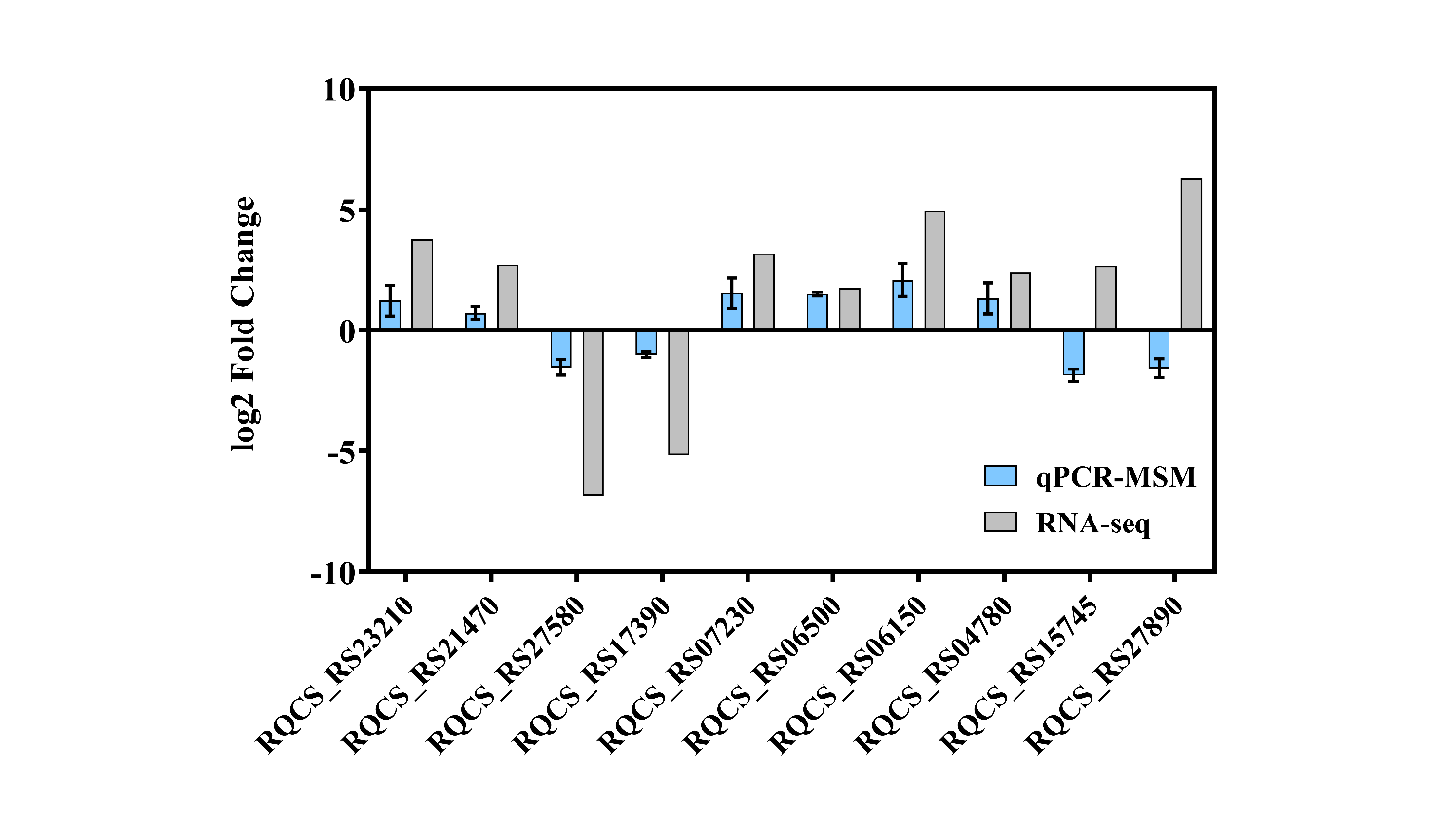


**Supplementary Figure 2.** RNA-seq profiles of strain RCBS9 surviving at 10℃ in MSM medium were verified by RT-qPCR

## Supplementary Tables

**Table S1. Gene primers used in RT-qPCR experiments.**

| Gene | Primer |  |
| --- | --- | --- |
| *RQCS_RS23210* | *FORWARD* | *GCATCGGTTCGTCGGCTTGG* |
|  | *REVERSE* | *GCGGATGTGGAAGAGCAGATCG* |
| *RQCS_RS21470* | *FORWARD* | *GACTCGACATCATCCACGCACTC* |
|  | *REVERSE* | *TCCGCCTCCGCCAAGATCG* |
| *RQCS_RS27580* | *FORWARD* | *ACCGACCGAGCCGTTGAGTG* |
|  | *REVERSE* | *CGACCAGTAGTGCGATGATCCTTC* |
| *RQCS_RS17390* | *FORWARD* | *TCAGAAGACACCCAACGAGATTGC* |
|  | *REVERSE* | *ATGGCTACGACGGCGAGGAG* |
| *RQCS_RS07230* | *FORWARD* | *ACGTCCACCAGGTCGCCAAG* |
|  | *REVERSE* | *AAGTGTCCGCCTGCCGAGAG* |
| *RQCS_RS06500* | *FORWARD* | *GCAGCGAGTTCCAGAAGATCACC* |
|  | *REVERSE* | *CGATGTCCTTGACGGCGATTCC* |
| *RQCS_RS06150* | *FORWARD* | *CCGACAGTCGCACCGCAATC* |
|  | *REVERSE* | *AGCACGAACGAACCACTGGAAC* |
| *RQCS_RS04780* | *FORWARD* | *CTCCACTACCTACGGCGTCAC* |
|  | *REVERSE* | *CTCGAAGTTCCACTGAATGTCTCC* |

**Table S2. Target genes and primer sequences selected for construction of recombinant bacteria.**

| Gene | enzyme | Primer |  |
| --- | --- | --- | --- |
| *RQCS_RS05830* | *sHsps* | *FORWARD* | *cgGAATTCgtgcttcgtttcgatcctttca* |
|  |  | *REVERSE* | *cccAAGCTTtcaggcttcgatctgtccttg* |
| *RQCS_RS06150* | *DPS* | *FORWARD* | *cgGAATTCatgtccgagttcaccgtaccg* |
|  |  | *REVERSE* | *cccAAGCTTtcagttctgacgcgcctcatt* |
| *RQCS_RS07390* | *GroEL* | *FORWARD* | *cgGAATTCatggcaaagatcatcgcgttcg* |
|  |  | *REVERSE* | *cccAAGCTTtcagaagtccatgccgccca* |
| *RQCS_RS15550* | *USP-1* | *FORWARD* | *cgGAATTCatgagtgcctaccggaccgt* |
|  |  | *REVERSE* | *cccAAGCTTtcagcgaacggtgtgcacga* |
| *BL21-RS07230* | *Cu/Zn-SOD* | *FORWARD* | *cgGAATTCatggcccccagcagcacc* |
|  |  | *REVERSE* | *cccAAGCTTtcagctcttggtgatgacacc* |
| *BL21-RS21470* | *USP-2* | *FORWARD* | *cgGAATTCatgagttcacgcgaacataacag* |
|  |  | *REVERSE* | *cccAAGCTTtcaggattcttcggggcgaac* |

**Table S3. Collation of genes for transcriptome results**-**Signal transduction and transcriptional regulation.**

| Gene ID | gene-name | Regulate | significant |
| --- | --- | --- | --- |
| *RQCS_RS03250* | *DESK* | *UP* | *yes* |
| *RQCS_RS01795* | *RegX3* | *UP* | *yes* |
| *RQCS_RS06550* | *NarL* | *UP* | *yes* |
| *RQCS_RS05215* | *NARL* | *UP* | *yes* |
| *RQCS_RS10520* | *mtrA* | *UP* | *yes* |
| *RQCS_RS02370* | *crp* | *UP* | *yes* |
| *RQCS_RS12920* | *lexA* | *UP* | *yes* |
| *RQCS_RS25690* | *lexA* | *UP* | *yes* |
| *RQCS_RS27890* | *OXYR* | *UP* | *yes* |
| *RQCS_RS16340* | *OXYR* | *UP* | *yes* |
| *RQCS_RS07795* | *OXYR* | *UP* | *yes* |
| *RQCS_RS16165* | *OXYR* | *UP* | *yes* |
| *RQCS_RS26225* | *soxR* | *UP* | *yes* |
| *RQCS_RS21000* | *soxR* | *UP* | *yes* |
| *RQCS_RS25715* | *ScbR* | *UP* | *yes* |
| *RQCS_RS20640* | *ScbR* | *UP* | *yes* |
| *RQCS_RS25660* | *GGDEF* | *UP* | *yes* |
| *RQCS_RS26240* | *GGDEF* | *UP* | *yes* |
| *RQCS_RS24070* | *adenylate/guanylate cyclase* | *UP* | *yes* |
| *RQCS_RS24040* | *alkaline phosphatase* | *UP* | *yes* |
| *RQCS_RS10985* | *alkaline phosphatase* | *UP* | *yes* |
| *RQCS_RS06595* | *EAL* | *UP* | *yes* |
| *RQCS_RS19015* | *Metallophos* | *UP* | *yes* |
| *RQCS_RS21415* | *GGDEF* | *Down* | *yes* |
| *RQCS_RS26550* | *EAL* | *Down* | *yes* |
| *RQCS_RS07190* | *adenylate/guanylate cyclase* | *Down* | *yes* |
| *RQCS_RS06950* | *GGDEF* | *Down* | *yes* |
| *RQCS_RS14625* | *GGDEF* | *Down* | *yes* |
| *RQCS_RS02670* | *GGDEF* | *Down* | *yes* |
| *RQCS_RS20075* | *EAL* | *Down* | *yes* |
| *RQCS_RS20070* | *EAL* | *Down* | *yes* |
| *RQCS_RS26545* | *GGDEF* | *Down* | *yes* |

**Table S4. Collation of genes for transcriptome results- Antioxidant stress**

| *Gene ID* | *gene-name* | *Regulate* | *significant* |
| --- | --- | --- | --- |
| *RQCS_RS00770* | *superoxide dismutase* | *UP* | *yes* |
| *RQCS_RS23375* | *superoxide dismutase* | *UP* | *yes* |
| *RQCS_RS07230* | *superoxide dismutase* | *UP* | *yes* |
| *RQCS_RS17105* | *peroxiredoxin* | *UP* | *yes* |
| *RQCS_RS15745* | *peroxiredoxin* | *UP* | *yes* |
| *RQCS_RS23210* | *Dyp-type peroxidase* | *UP* | *yes* |
| *RQCS_RS25130* | *peroxidase* | *UP* | *yes* |
| *RQCS_RS04780* | *glutathione peroxidase* | *UP* | *yes* |
| *RQCS_RS01175* | *hypothetical protein* | *UP* | *yes* |
| *RQCS_RS02455* | *ABC transporter substrate-binding protein* | *UP* | *yes* |
| *RQCS_RS02470* | *ABC transporter ATP-binding protein* | *UP* | *yes* |
| *RQCS_RS22600* | *ABC transporter substrate-binding protein* | *UP* | *yes* |
| *RQCS_RS23805* | *ABC transporter permease* | *UP* | *yes* |
| *RQCS_RS02615* | *ABC transporter substrate-binding protein* | *UP* | *yes* |
| *RQCS_RS07070* | *ABC transporter ATP-binding protein* | *UP* | *yes* |
| *RQCS_RS00260* | *ABC transporter substrate-binding protein* | *UP* | *yes* |
| *RQCS_RS15525* | *MBL fold metallo-hydrolase* | *UP* | *yes* |
| *RQCS_RS22340* | *4,4'-diapolycopene aldehyde oxidase* | *UP* | *yes* |
| *RQCS_RS16635* | *phytoene/squalene synthase family protein* | *UP* | *no* |
| *RQCS_RS12975* | *peroxiredoxin* | *Down* | *yes* |
| *RQCS_RS27580* | *catalase* | *Down* | *yes* |
| *RQCS_RS05700* | *catalase* | *Down* | *yes* |
| *RQCS_RS02195* | *catalase* | *Down* | *yes* |
| *RQCS_RS18125* | *thioredoxin-dependent thiol peroxidase* | *Down* | *yes* |
| *RQCS_RS28675* | *Dyp-type peroxidase* | *Down* | *yes* |
| *RQCS_RS19280* | *glutathione S-transferase C-terminal domain-containing protein* | *Down* | *yes* |
| *RQCS_RS05445* | *S-(hydroxymethyl)glutathione dehydrogenase* | *Down* | *yes* |
| *RQCS_RS19175* | *Deaminated glutathione amidase* | *Down* | *yes* |
| *RQCS_RS27755* | *S-(hydroxymethyl)glutathione dehydrogenase* | *Down* | *yes* |
| *RQCS_RS16625* | *phytoene desaturase family protein* | *Down* | *no* |

**Table S5. Collation of genes for transcriptome results- Maintenance and repair of genes.**

| *Gene ID* | *gene-name* | *Regulate* | *significant* |
| --- | --- | --- | --- |
| *RQCS_RS06150* | *DPS* | *UP* | *yes* |
| *RQCS_RS27680* | *DNA alkylation repair protein* | *UP* | *yes* |
| *RQCS_RS00625* | *RNase_H superfamily* | *UP* | *yes* |
| *RQCS_RS00010* | *dnaN* | *UP* | *yes* |
| *RQCS_RS28015* | *dnaB* | *UP* | *yes* |
| *RQCS_RS10775* | *ATP-dependent DNA helicase* | *UP* | *yes* |
| *RQCS_RS27595* | *NA polymerase recycling motor ATPase HelR* | *UP* | *yes* |
| *RQCS_RS27595* | *NA polymerase recycling motor ATPase HelR* | *UP* | *yes* |
| *RQCS_RS12920* | *lexA* | *UP* | *yes* |
| *RQCS_RS25690* | *lexA* | *UP* | *yes* |
| *RQCS_RS09800* | *exodeoxyribonuclease III* | *UP* | *yes* |
| *RQCS_RS24615* | *ribonuclease H* | *UP* | *yes* |
| *RQCS_RS10540* | *ribosome-associated translation inhibitor RaiA* | *UP* | *yes* |
| *RQCS_RS03860* | *UvrD* | *UP* | *yes* |
| *RQCS_RS10775* | *UvrD* | *UP* | *yes* |
| *RQCS_RS03855* | *recC* | *UP* | *yes* |
| *RQCS_RS11545* | *recG* | *UP* | *yes* |
| *RQCS_RS12805* | *recA* | *UP* | *yes* |
| *RQCS_RS00020* | *recF* | *UP* | *yes* |
| *RQCS_RS12810* | *recX* | *UP* | *yes* |
| *RQCS_RS23915* | *DinB* | *UP* | *yes* |
| *RQCS_RS16480* | *DNA polymerase IV* | *UP* | *yes* |
| *RQCS_RS03325* | *MUTY* | *UP* | *yes* |
| *RQCS_RS04140* | *YcaQ* | *UP* | *yes* |
| *RQCS_RS19765* | *MUG* | *UP* | *yes* |
| *RQCS_RS11525* | *UNG* | *UP* | *yes* |
| *RQCS_RS18145* | *type I polyketide synthase* | *Down* | *yes* |
| *RQCS_RS17255* | *dnaG* | *Down* | *yes* |
| *RQCS_RS15630* | *polA* | *Down* | *yes* |
| *RQCS_RS13900* | *coaBC* | *Down* | *yes* |
| *RQCS_RS07340* | *DNA-binding transcriptional regulator* | *Down* | *yes* |
| *RQCS_RS00045* | *gyrB* | *Down* | *yes* |
| *RQCS_RS17330* | *recO* | *Down* | *yes* |
| *RQCS_RS21610* | *ATP-dependent DNA ligase* | *Down* | *yes* |
| *RQCS_RS01840* | *ATP-dependent DNA ligase* | *Down* | *yes* |
| *RQCS_RS22250* | *DEAD/DEAH box helicase* | *Down* | *yes* |
| *RQCS_RS23760* | *ligD* | *Down* | *yes* |
| *RQCS_RS13030* | *metal-dependent transcriptional regulator* | *Down* | *yes* |
| *RQCS_RS12715* | *DNA translocase FtsK* | *Down* | *yes* |
| *RQCS_RS02380* | *nth* | *Down* | *yes* |
| *RQCS_RS21410* | *DNA-formamidopyrimidine glycosylase family protein* | *Down* | *yes* |
| *RQCS_RS11315* | *ligA* | *Down* | *yes* |
| *RQCS_RS15375* | *DNA-3-methyladenine glycosylase* | *Down* | *yes* |
| *RQCS_RS21910* | *deoxyribodipyrimidine photo-lyase* | *Down* | *yes* |
| *RQCS_RS02535* | *topA* | *Down* | *yes* |
| *RQCS_RS17360* | *dinB* | *Down* | *yes* |
| *RQCS_RS17595* | *holA* | *Down* | *yes* |
| *RQCS_RS21280* | *pcrA* | *Down* | *yes* |
| *RQCS_RS08620* | *DNA alkylation repair protein* | *Down* | *yes* |
| *RQCS_RS01900* | *DNA polymerase III subunit gamma and tau* | *Down* | *yes* |
| *RQCS_RS09665* | *error-prone DNA polymerase* | *Down* | *yes* |
| *RQCS_RS00050* | *gyrA* | *Down* | *yes* |
| *RQCS_RS02540* | *DNA polymerase III subunit delta* | *Down* | *yes* |
| *RQCS_RS15325* | *recN* | *Down* | *yes* |
| *RQCS_RS03385* | *disA* | *Down* | *yes* |
| *RQCS_RS23765* | *ATP-dependent DNA ligase* | *Down* | *yes* |
| *RQCS_RS14535* | *DEAD/DEAH box helicase* | *Down* | *yes* |
| *RQCS_RS19170* | *DEAD/DEAH box helicase* | *Down* | *yes* |
| *RQCS_RS10665* | *DEAD/DEAH box helicase* | *Down* | *yes* |
| *RQCS_RS12935* | *hrpA* | *Down* | *yes* |
| *RQCS_RS14525* | *DEAD/DEAH box helicase* | *Down* | *yes* |
| *RQCS_RS13000* | *DEAD/DEAH box helicase* | *Down* | *yes* |
| *RQCS_RS11090* | *DEAD/DEAH box helicase* | *Down* | *yes* |
| *RQCS_RS02205* | *DEAD/DEAH box helicase* | *Down* | *yes* |
| *RQCS_RS15580* | *rpsA* | *Down* | *yes* |
| *RQCS_RS12835* | *miaB* | *Down* | *yes* |
| *RQCS_RS09380* | *sigma-70 family RNA polymerase sigma factor* | *Down* | *yes* |
| *RQCS_RS11485* | *RNA degradosome polyphosphate kinase* | *Down* | *yes* |
| *RQCS_RS17030* | *ribonuclease* | *Down* | *yes* |
| *RQCS_RS09360* | *sigma-70 family RNA polymerase sigma factor* | *Down* | *yes* |
| *RQCS_RS06120* | *SigB/SigF/SigG family RNA polymerase sigma factor* | *Down* | *yes* |
| *RQCS_RS03185* | *lysS* | *Down* | *yes* |
| *RQCS_RS04975* | *trmB* | *Down* | *yes* |
| *RQCS_RS18415* | *argS* | *Down* | *yes* |
| *RQCS_RS19490* | *sigE* | *Down* | *yes* |
| *RQCS_RS15370* | *tyrS* | *Down* | *yes* |
| *RQCS_RS15370* | *tyrS* | *Down* | *yes* |
| *RQCS_RS12130* | *proline--tRNA ligase* | *Down* | *yes* |
| *RQCS_RS01730* | *tgt* | *Down* | *yes* |
| *RQCS_RS11835* | *rlmN* | *Down* | *yes* |
| *RQCS_RS19070* | *sigma-70 family RNA polymerase sigma factor* | *Down* | *yes* |
| *RQCS_RS14650* | *RNA polymerase-binding protein RbpA* | *Down* | *yes* |
| *RQCS_RS17745* | *RNA polymerase-binding protein RbpA* | *Down* | *yes* |
| *RQCS_RS08340* | *rplK* | *Down* | *yes* |
| *RQCS_RS08340* | *rplK* | *Down* | *yes* |
| *RQCS_RS19055* | *23S ribosomal RNA* | *Down* | *yes* |
| *RQCS_RS11305* | *mnmA* | *Down* | *yes* |
| *RQCS_RS11365* | *gatB* | *Down* | *yes* |
| *RQCS_RS03405* | *carD* | *Down* | *yes* |
| *RQCS_RS03420* | *cysS* | *Down* | *yes* |
| *RQCS_RS15360* | *23S ribosomal RNA* | *Down* | *yes* |
| *RQCS_RS18290* | *24S ribosomal RNA* | *Down* | *yes* |
| *RQCS_RS09325* | *tsaD* | *Down* | *yes* |
| *RQCS_RS13175* | *methyltransferase* | *Down* | *yes* |
| *RQCS_RS13100* | *RNA polymerase sigma factor* | *Down* | *yes* |
| *RQCS_RS18180* | *rph* | *Down* | *yes* |
| *RQCS_RS00930* | *serS* | *Down* | *yes* |
| *RQCS_RS11580* | *rnc* | *Down* | *yes* |
| *RQCS_RS11435* | *gltX* | *Down* | *yes* |
| *RQCS_RS13935* | *transcription antitermination factor NusB* | *Down* | *yes* |
| *RQCS_RS13655* | *methyltransferase* | *Down* | *yes* |
| *RQCS_RS28740* | *leuS* | *Down* | *yes* |
| *RQCS_RS12855* | *miaA* | *Down* | *yes* |
| *RQCS_RS23005* | *dusB* | *Down* | *yes* |
| *RQCS_RS12250* | *truB* | *Down* | *yes* |
| *RQCS_RS13930* | *fmt* | *Down* | *yes* |
| *RQCS_RS10790* | *nudC* | *Down* | *yes* |
| *RQCS_RS00320* | *lysX* | *Down* | *yes* |
| *RQCS_RS20945* | *metG* | *Down* | *yes* |
| *RQCS_RS07635* | *RNA polymerase sigma factor SigF* | *Down* | *yes* |
| *RQCS_RS14835* | *RNA helicase* | *Down* | *yes* |
| *RQCS_RS10610* | *sigma-70 family RNA polymerase sigma factor* | *Down* | *yes* |
| *RQCS_RS12705* | *ribonuclease* | *Down* | *yes* |
| *RQCS_RS03425* | *rlmB* | *Down* | *yes* |
| *RQCS_RS02570* | *23S ribosomal RNA* | *Down* | *yes* |
| *RQCS_RS22295* | *sigma-70 family RNA polymerase sigma factor* | *Down* | *yes* |
| *RQCS_RS02635* | *23S ribosomal RNA* | *Down* | *yes* |
| *RQCS_RS19060* | *16S ribosomal RNA* | *Down* | *yes* |
| *RQCS_RS15365* | *16S ribosomal RNA* | *Down* | *yes* |
| *RQCS_RS14985* | *mshC* | *Down* | *yes* |
| *RQCS_RS17455* | *methyltransferase* | *Down* | *yes* |
| *RQCS_RS09055* | *rplB* | *Down* | *yes* |
| *RQCS_RS13365* | *thrS* | *Down* | *yes* |
| *RQCS_RS11645* | *RNA-binding protein* | *Down* | *yes* |
| *RQCS_RS09315* | *tsaB* | *Down* | *yes* |
| *RQCS_RS20740* | *pth* | *Down* | *yes* |
| *RQCS_RS12035* | *RNA polymerase sigma factor* | *Down* | *yes* |
| *RQCS_RS27420* | *RNA polymerase sigma factor* | *Down* | *yes* |
| *RQCS_RS15355* | *rrf* | *Down* | *yes* |
| *RQCS_RS15480* | *RNA methyltransferase* | *Down* | *yes* |
| *RQCS_RS28345* | *amidase* | *Down* | *yes* |
| *RQCS_RS18295* | *amidase* | *Down* | *yes* |
| *RQCS_RS12600* | *amidase* | *Down* | *yes* |
| *RQCS_RS13980* | *rapZ* | *Down* | *yes* |
| *RQCS_RS13530* | *hisS* | *Down* | *yes* |
| *RQCS_RS22015* | *RNA polymerase sigma factor SigD* | *Down* | *yes* |
| *RQCS_RS02565* | *16S ribosomal RNA* | *Down* | *yes* |
| *RQCS_RS02630* | *17S ribosomal RNA* | *Down* | *yes* |
| *RQCS_RS16660* | *exoribonuclease* | *Down* | *yes* |
| *RQCS_RS22850* | *RQCS_RS22850* | *Down* | *yes* |
| *RQCS_RS16490* | *ileS* | *Down* | *yes* |
| *RQCS_RS15335* | *methyltransferase* | *Down* | *yes* |
| *RQCS_RS15325* | *recN* | *Down* | *yes* |

**Table S6. Collation of genes for transcriptome results-Cell membrane.**

| *Gene ID* | *gene-name* | *Regulate* | *significant* |
| --- | --- | --- | --- |
| *RQCS_RS26000* | *alkane 1-monooxygenase* | *UP* | *yes* |
| *RQCS_RS17350* | *FabG* | *UP* | *yes* |
| *RQCS_RS23250* | *SDR family oxidoreductase* | *UP* | *yes* |
| *RQCS_RS24765* | *fabG* | *UP* | *yes* |
| *RQCS_RS00570* | *4-coumarate--CoA ligase 2* | *UP* | *yes* |
| *RQCS_RS01885* | *long-chain fatty acid--CoA ligase* | *UP* | *yes* |
| *RQCS_RS01885* | *long-chain fatty acid--CoA ligase* | *UP* | *yes* |
| *RQCS_RS16695* | *long-chain fatty acid--CoA ligase* | *UP* | *yes* |
| *RQCS_RS24695* | *4-coumarate--CoA ligase 2* | *UP* | *yes* |
| *RQCS_RS24695* | *4-coumarate--CoA ligase 3* | *UP* | *yes* |
| *RQCS_RS19190* | *long-chain fatty acid--CoA ligase* | *UP* | *yes* |
| *RQCS_RS00995* | *acyl-CoA dehydrogenase* | *UP* | *yes* |
| *RQCS_RS26745* | *enoyl-CoA hydratase* | *UP* | *yes* |
| *RQCS_RS23250* | *SDR family oxidoreductase* | *UP* | *yes* |
| *RQCS_RS00990* | *Beta-ketothiolase BktB* | *UP* | *yes* |
| *RQCS_RS21705* | *enoyl-CoA hydratase* | *UP* | *yes* |
| *RQCS_RS17350* | *3-oxoacyl-ACP reductase* | *UP* | *yes* |
| *RQCS_RS23535* | *acyl-CoA dehydrogenase* | *UP* | *yes* |
| *RQCS_RS20850* | *acetyl-CoA acetyltransferase* | *UP* | *yes* |
| *RQCS_RS04200* | *steroid 3-ketoacyl-CoA thiolase* | *UP* | *yes* |
| *RQCS_RS00995* | *acyl-CoA dehydrogenase* | *UP* | *yes* |
| *RQCS_RS26745* | *enoyl-CoA hydratase* | *UP* | *yes* |
| *RQCS_RS26530* | *Alpha-ketoglutaric semialdehyde dehydrogenase* | *UP* | *yes* |
| *RQCS_RS00990* | *Beta-ketothiolase BktB* | *UP* | *yes* |
| *RQCS_RS21705* | *enoyl-CoA hydratase* | *UP* | *yes* |
| *RQCS_RS23535* | *acyl-CoA dehydrogenase* | *UP* | *yes* |
| *RQCS_RS27145* | *cetyl-CoA C-acetyltransferase* | *UP* | *yes* |
| *RQCS_RS20850* | *acetyl-CoA acetyltransferase* | *UP* | *yes* |
| *RQCS_RS04200* | *steroid 3-ketoacyl-CoA thiolas* | *UP* | *yes* |
| *RQCS_RS20035* | *acetyl-CoA C-acetyltransferase* | *UP* | *yes* |
| *RQCS_RS17160* | *NDMA-dependent alcohol dehydrogenase* | *UP* | *yes* |
| *RQCS_RS22455* | *enoyl-CoA hydratase family protein* | *UP* | *yes* |
| *RQCS_RS24920* | *acyl-CoA dehydrogenase* | *UP* | *yes* |
| *RQCS_RS05050* | *aldehyde dehydrogenas* | *UP* | *yes* |
| *RQCS_RS24685* | *Steroid 3-ketoacyl-CoA thiolase FadA6* | *UP* | *yes* |
| *RQCS_RS23275* | *acyl-CoA dehydrogenase* | *UP* | *yes* |
| *RQCS_RS04250* | *enoyl-CoA hydratas* | *UP* | *yes* |
| *RQCS_RS24530* | *enoyl-CoA hydratase-related protein* | *UP* | *yes* |
| *RQCS_RS13230* | *3-oxo-5,6-dehydrosuberyl-CoA thiolase* | *UP* | *yes* |
| *RQCS_RS19245* | *enoyl-CoA hydratase* | *UP* | *yes* |
| *RQCS_RS28430* | *acyl-CoA dehydrogenase* | *UP* | *yes* |
| *RQCS_RS25175* | *acetyl-CoA C-acetyltransferase* | *UP* | *yes* |
| *RQCS_RS24295* | *steroid 3-ketoacyl-CoA thiolas* | *UP* | *yes* |
| *RQCS_RS24285* | *lipid-transfer protein* | *UP* | *yes* |
| *RQCS_RS24755* | *acetyl-CoA C-acetyltransferase* | *UP* | *yes* |
| *RQCS_RS26930* | *lipid-transfer protein* | *UP* | *yes* |
| *RQCS_RS00570* | *4-coumarate--CoA ligase* | *UP* | *yes* |
| *RQCS_RS04175* | *enoyl-CoA hydratase* | *UP* | *yes* |
| *RQCS_RS01920* | *acetyl-CoA C-acetyltransferase* | *UP* | *yes* |
| *RQCS_RS01885* | *long-chain fatty acid--CoA ligase* | *UP* | *yes* |
| *RQCS_RS18550* | *enoyl-CoA hydratase* | *UP* | *yes* |
| *RQCS_RS16695* | *long-chain fatty acid--CoA ligase* | *UP* | *yes* |
| *RQCS_RS01165* | *Sterol carrier protein 2* | *UP* | *yes* |
| *RQCS_RS22305* | *acyl-CoA dehydrogenase* | *UP* | *yes* |
| *RQCS_RS02020* | *steroid 3-ketoacyl-CoA thiolase* | *UP* | *yes* |
| *RQCS_RS24695* | *4-coumarate--CoA ligase 2* | *UP* | *yes* |
| *RQCS_RS08605* | *FAD-dependent oxidoreductase* | *UP* | *yes* |
| *RQCS_RS26175* | *Long-chain-fatty-acid--CoA ligase* | *UP* | *yes* |
| *RQCS_RS26000* | *alkane 1-monooxygenase* | *UP* | *yes* |
| *RQCS_RS19190* | *long-chain fatty acid--CoA ligase* | *UP* | *yes* |
| *RQCS_RS06870* | *Steroid 3-ketoacyl-CoA thiolase FadA6* | *UP* | *yes* |
| *RQCS_RS15870* | *aldehyde dehydrogenase* | *UP* | *yes* |
| *RQCS_RS26465* | *3-hydroxyacyl-CoA dehydrogenase* | *UP* | *yes* |
| *RQCS_RS10580* | *fatty acid desaturase* | *Down* | *yes* |
| *RQCS_RS03710* | *alkane 1-monooxygenase* | *Down* | *yes* |
| *RQCS_RS24190* | *fatty acid desaturas* | *Down* | *yes* |
| *RQCS_RS23000* | *acyl-ACP desaturase* | *Down* | *yes* |
| *RQCS_RS18020* | *acyl-CoA desaturase* | *Down* | *yes* |
| *RQCS_RS10575* | *acyl-CoA desaturase* | *Down* | *yes* |
| *RQCS_RS11430* | *alkane 1-monooxygenase* | *Down* | *yes* |
| *RQCS_RS10490* | *alkane 2-monooxygenase* | *Down* | *yes* |
| *RQCS_RS17130* | *ACP S-malonyltransferase* | *Down* | *yes* |
| *RQCS_RS18145* | *type I polyketide synthase* | *Down* | *yes* |
| *RQCS_RS08935* | *acetyl/propionyl/methylcrotonyl-CoA* | *Down* | *yes* |
| *RQCS_RS03710* | *alkane 1-monooxygenase* | *Down* | *yes* |
| *RQCS_RS18900* | *long-chain fatty acid--CoA ligase* | *Down* | *yes* |
| *RQCS_RS03725* | *FAD-dependent oxidoreductase* | *Down* | *yes* |
| *RQCS_RS12285* | *alcohol dehydrogenase* | *Down* | *yes* |
| *RQCS_RS11370* | *NAD(P)-dependent alcohol dehydrogenase* | *Down* | *yes* |
| *RQCS_RS10895* | *acyl-CoA dehydrogenas* | *Down* | *yes* |
| *RQCS_RS12145* | *acyl-CoA dehydrogenas* | *Down* | *yes* |
| *RQCS_RS18560* | *enoyl-CoA hydratase* | *Down* | *yes* |
| *RQCS_RS05145* | *acetyl-CoA C-acetyltransferase* | *Down* | *yes* |
| *RQCS_RS11430* | *alkane 1-monooxygenase* | *Down* | *yes* |
| *RQCS_RS08540* | *enoyl-CoA hydratase* | *Down* | *yes* |
| *RQCS_RS26115* | *acetyl-CoA C-acyltransferase* | *Down* | *yes* |
| *RQCS_RS10490* | *alkane 1-monooxygenase* | *Down* | *yes* |
| *RQCS_RS03055* | *enoyl-CoA hydratas* | *Down* | *yes* |
| *RQCS_RS16990* | *NDMA-dependent alcohol dehydrogenase* | *Down* | *yes* |
| *RQCS_RS26150* | *acyl-CoA dehydrogenas* | *Down* | *yes* |
| *RQCS_RS01925* | *3-hydroxyacyl-CoA dehydrogenase* | *Down* | *yes* |
| *RQCS_RS02580* | *aldehyde dehydrogenase* | *Down* | *yes* |
| *RQCS_RS23975* | *enoyl-CoA hydratase* | *Down* | *yes* |
| *RQCS_RS08055* | *acyl-CoA dehydrogenase* | *Down* | *yes* |
| *RQCS_RS23870* | *3-hydroxyacyl-CoA dehydrogenase* | *Down* | *yes* |

**Table S7. The top 20 genes of high degree obtained from protein-protein interaction (PPI) network analysis.**

| *Gene ID* | *gene-name* | *Regulate* | *significant* |
| --- | --- | --- | --- |
| *RQCS_RS03530* | *3-oxo-4,17-pregnadiene-20-carboxyl-CoA hydratase* | *UP* | *yes* |
| *RQCS_RS24665* | *MaoC family dehydratase* | *UP* | *yes* |
| *RQCS_RS08945* | *Mesaconyl-CoA hydratase* | *UP* | *yes* |
| *RQCS_RS24280* | *Peroxisomal multifunctional enzyme type 2* | *UP* | *yes* |
| *RQCS_RS03660* | *3-hydroxyacyl-thioester dehydratase* | *UP* | *yes* |
| *RQCS_RS24140* | *Peroxisomal multifunctional enzyme type 2* | *UP* | *yes* |
| *RQCS_RS08705* | *Peroxisomal multifunctional enzyme type 2* | *UP* | *yes* |
| *RQCS_RS04350* | *Acyl-CoA dehydrogenase* | *UP* | *yes* |
| *RQCS_RS24920* | *Acyl-CoA dehydrogenase* | *UP* | *yes* |
| *RQCS_RS04005* | *Acyl-CoA dehydrogenase* | *UP* | *yes* |
| *RQCS_RS26460* | *Acyl-CoA dehydrogenase* | *UP* | *yes* |
| *RQCS_RS04175* | *Acyl-CoA dehydrogenase* | *UP* | *yes* |
| *RQCS_RS11530* | *Acyl-CoA dehydrogenase* | *UP* | *yes* |
| *RQCS_RS11235* | *Acyl-CoA dehydrogenase* | *UP* | *yes* |
| *RQCS_RS21705* | *Acyl-CoA dehydrogenase* | *UP* | *yes* |
| *RQCS_RS22455* | *Acyl-CoA dehydrogenase* | *UP* | *yes* |
| *RQCS_RS04250* | *Acyl-CoA dehydrogenase* | *UP* | *yes* |
| *RQCS_RS19245* | *Acyl-CoA dehydrogenase* | *UP* | *yes* |
| *RQCS_RS13225* | *3-hydroxyacyl-CoA dehydrogenase* | *UP* | *yes* |
| *RQCS_RS26480* | *phenylacetic acid degradation bifunctional protein* | *UP* | *yes* |

**Table S8. Collation of genes for transcriptome results-Cell wall**

| *Gene ID* | *gene-name* | *Regulate* | *significant* |
| --- | --- | --- | --- |
| *RQCS_RS03065* | *D-alanyl-D-alanine carboxypeptidase* | *UP* | *yes* |
| *RQCS_RS15015* | *undecaprenyl-diphosphate phosphatase* | *UP* | *yes* |
| *RQCS_RS00155* | *RQCS_RS00155* | *UP* | *yes* |
| *RQCS_RS16550* | *UDP-N-acetylmuramoyl-L-alanine--D-glutamate ligase* | *UP* | *yes* |
| *RQCS_RS02050* | *MurT ligase domain-containing protein* | *UP* | *yes* |
| *RQCS_RS25985* | *Serine racemase* | *UP* | *yes* |
| *RQCS_RS26080* | *amino acid racemase* | *UP* | *yes* |
| *RQCS_RS22785* | *aspartate/glutamate racemase family protein* | *UP* | *yes* |
| *RQCS_RS07750* | *UDP-N-acetylmuramate dehydrogenase* | *Down* | *yes* |
| *RQCS_RS18300* | *UDP-N-acetylglucosamine 1-carboxyvinyltransferase* | *Down* | *yes* |
| *RQCS_RS18540* | *phospho-N-acetylmuramoyl-pentapeptide-transferase* | *Down* | *yes* |
| *RQCS_RS17325* | *isoprenyl transferase* | *Down* | *yes* |
| *RQCS_RS11505* | *D-alanine--D-alanine ligase family protein* | *Down* | *yes* |
| *RQCS_RS16560* | *UDP-N-acetylmuramoyl-tripeptide--D-alanyl-D-alanine ligase* | *Down* | *yes* |
| *RQCS_RS16565* | *UDP-N-acetylmuramoyl-L-alanyl-D-glutamate--2,6-diaminopimelate ligase* | *Down* | *yes* |
| *RQCS_RS13850* | *aspartate carbamoyltransferase catalytic subunit* | *Down* | *yes* |
| *RQCS_RS08610* | *aspartate/glutamate racemase family protein* | *Down* | *yes* |
| *RQCS_RS09300* | *alanine racemase* | *Down* | *yes* |

**Table S9. Collation of genes for transcriptome results-Stress-regulated protein.**

| *Gene ID* | *gene-name* | *Regulate* | *significant* |
| --- | --- | --- | --- |
| *RQCS_RS15550* | *USP* | *UP* | *yes* |
| *RQCS_RS21470* | *USP* | *UP* | *yes* |
| *RQCS_RS21180* | *USP* | *UP* | *yes* |
| *RQCS_RS05710* | *USP* | *UP* | *yes* |
| *RQCS_RS22860* | *USP* | *UP* | *yes* |
| *RQCS_RS21460* | *USP* | *UP* | *yes* |
| *RQCS_RS15975* | *USP* | *UP* | *yes* |
| *RQCS_RS20735* | *GSP* | *UP* | *yes* |
| *RQCS_RS08600* | *envelope stress response protein* | *UP* | *yes* |
| *RQCS_RS06235* | *envelope stress response protein* | *UP* | *yes* |
| *RQCS_RS08600* | *envelope stress response protein* | *UP* | *yes* |
| *RQCS_RS06500* | *dnaK* | *UP* | *yes* |
| *RQCS_RS23920* | *htpG* | *UP* | *yes* |
| *RQCS_RS24330* | *Hsp90 ATPase* | *UP* | *yes* |
| *RQCS_RS15960* | *Hsp91 ATPase* | *UP* | *yes* |
| *RQCS_RS25755* | *Hsp92 ATPase* | *UP* | *yes* |
| *RQCS_RS26095* | *Hsp93 ATPase* | *UP* | *yes* |
| *RQCS_RS28005* | *sHsps* | *UP* | *yes* |
| *RQCS_RS05830* | *sHsps* | *UP* | *yes* |
| *RQCS_RS06160* | *HSPR* | *UP* | *yes* |
| *RQCS_RS06515* | *HSPR* | *UP* | *yes* |
| *RQCS_RS06505* | *grpE* | *UP* | *yes* |
| *RQCS_RS06510* | *dnaJ* | *UP* | *yes* |
| *RQCS_RS07390* | *groL\GroEL* | *UP* | *yes* |
| *RQCS_RS09345* | *groES* | *UP* | *yes* |
| *RQCS_RS17820* | *CLPP2* | *UP* | *yes* |
| *RQCS_RS17815* | *CLPP1* | *UP* | *yes* |
| *RQCS_RS28415* | *CLPP3* | *UP* | *yes* |
| *RQCS_RS28410* | *CLPP4* | *UP* | *yes* |
| *RQCS_RS22080* | *peptidylprolyl isomerase* | *UP* | *yes* |
| *RQCS_RS13520* | *peptidylprolyl isomerase* | *UP* | *yes* |
| *RQCS_RS13515* | *peptidylprolyl isomerase* | *UP* | *yes* |
| *RQCS_RS08915* | *envelope stress response protein* | *Down* | *yes* |
| *RQCS_RS17515* | *envelope stress response protein* | *Down* | *yes* |
| *RQCS_RS27980* | *ClpB 2* | *Down* | *yes* |
| *RQCS_RS18205* | *clpS* | *Down* | *yes* |
| *RQCS_RS22635* | *CLPC* | *Down* | *yes* |
| *RQCS_RS06720* | *clpB* | *Down* | *yes* |
| *RQCS_RS08135* | *CLPC1* | *Down* | *yes* |
| *RQCS_RS06395* | *HSP70* | *Down* | *yes* |
| *RQCS_RS09330* | *HSP70* | *Down* | *yes* |
| *RQCS_RS06460* | *HSP70* | *Down* | *yes* |
| *RQCS_RS00090* | *peptidylprolyl isomerase* | *Down* | *yes* |

**Table S10. Collation of genes for transcriptome results- Anti-Osmotic Stress**

| *Gene ID* | *gene-name* | *Regulate* | *significant* |
| --- | --- | --- | --- |
| *RQCS_RS21085* | *mscL* | *UP* | *yes* |
| *RQCS_RS22055* | *aquaporin* | *UP* | *yes* |
| *RQCS_RS17570* | *aquaporin* | *UP* | *yes* |
| *RQCS_RS05655* | *PROP* | *UP* | *yes* |
| *RQCS_RS03895* | *PROP* | *UP* | *yes* |
| *RQCS_RS19340* | *OPUCC* | *UP* | *yes* |
| *RQCS_RS19335* | *OPUCC* | *UP* | *yes* |
| *RQCS_RS19350* | *OPUCB* | *UP* | *yes* |
| *RQCS_RS23515* | *OSMX* | *UP* | *yes* |
| *RQCS_RS26270* | *betB* | *UP* | *yes* |
| *RQCS_RS23500* | *OpuBB* | *UP* | *yes* |
| *RQCS_RS23510* | *OpuBB* | *UP* | *yes* |
| *RQCS_RS19345* | *OpuBA* | *UP* | *yes* |
| *RQCS_RS19350* | *OPUCB* | *UP* | *yes* |
| *RQCS_RS23505* | *OsmV* | *UP* | *yes* |
| *RQCS_RS28385* | *MalE* | *UP* | *yes* |
| *RQCS_RS19435* | *MalE* | *UP* | *yes* |
| *RQCS_RS28375* | *SugB* | *UP* | *yes* |
| *RQCS_RS17200* | *MalF* | *UP* | *yes* |
| *RQCS_RS28380* | *MalF* | *UP* | *yes* |
| *RQCS_RS16310* | *malto-oligosyltrehalose synthase* | *UP* | *yes* |
| *RQCS_RS00595* | *Trehalose 2-sulfotransferase* | *UP* | *yes* |
| *RQCS_RS03500* | *trehalose-phosphatase* | *Down* | *yes* |
| *RQCS_RS13555* | *trehalose-phosphatase* | *Down* | *yes* |
| *RQCS_RS16400* | *malto-oligosyltrehalose trehalohydrolase* | *Down* | *yes* |
| *RQCS_RS20965* | *betaine/carnitine/choline family transporter* | *Down* | *yes* |
| *RQCS_RS20965* | *betaine/carnitine/choline family transporter* | *Down* | *yes* |
| *RQCS_RS02705* | *betaine/carnitine/choline family transporter* | *Down* | *yes* |
| *RQCS_RS17655* | *PROP* | *Down* | *yes* |
| *RQCS_RS23630* | *PROP* | *Down* | *yes* |
| *RQCS_RS02705* | *betaine/carnitine/choline family transporter* | *Down* | *yes* |
| *RQCS_RS17215* | *betaine/carnitine/choline family transporter* | *Down* | *yes* |
